# Electrical in-situ characterisation of interface stabilised organic thin-film transistors

**DOI:** 10.1002/pssr.201510169

**Published:** 2015-07-14

**Authors:** Bernd Striedinger, Alexander Fian, Andreas Petritz, Roman Lassnig, Adolf Winkler, Barbara Stadlober

**Affiliations:** 1 Joanneum Research Forschungsgesellschaft mbH, MATERIALS-Institute for Surface Technologies and Photonics, Franz-Pichler Straße 30, 8160 Weiz, Austria; 2 Graz University of Technology, Institute of Solid State Physics, Petersgasse 16, 8010 Graz, Austria

**Keywords:** in-situ characterisation, pentacene, organic thin-film transistors, polymer dielectric, norbornene

## Abstract

We report on the electrical in-situ characterisation of organic thin film transistors under high vacuum conditions. Model devices in a bottom-gate/bottom-contact (coplanar) configuration are electrically characterised in-situ, monolayer by monolayer (ML), while the organic semiconductor (OSC) is evaporated by organic molecular beam epitaxy (OMBE). Thermal SiO_2_ with an optional polymer interface stabilisation layer serves as the gate dielectric and pentacene is chosen as the organic semiconductor. The evolution of transistor parameters is studied on a bi-layer dielectric of a 150 nm of SiO_2_ and 20 nm of poly((±)endo,exo-bicyclo[2.2.1]hept-5-ene-2,3-dicarboxylic acid, diphenylester) (PNDPE) and compared to the behaviour on a pure SiO_2_ dielectric. The thin layer of PNDPE, which is an intrinsically photo-patternable organic dielectric, shows an excellent stabilisation performance, significantly reducing the calculated interface trap density at the OSC/dielectric interface up to two orders of magnitude, and thus remarkably improving the transistor performance.

## 1 Introduction

Electronic devices based on organic materials are generally expected to be of great future importance. In order to implement organic thin film transistors (OTFTs) into useful, market-ready applications and circuits, one has to gain precise control over the electrical characteristics and their reproducibility which in turn requires to precisely control the interface properties of functional layers, i.e. the dielectric layer and the organic semiconductor (OSC) layer. It is generally accepted that the majority of the current in an OTFT flows in the very first monolayers (ML) of the OSC layer [1–4]. Thus it is not surprising that the properties of the dielectric/OSC-interface are critical to the device performance, since they strongly affect the growth mode of the OSC as well as charge trapping at or near the interface. Accordingly, key device parameters such as the field-effect mobility *μ*, the threshold-voltage *V*_th_, the onset-voltage *V*_on_, etc. are critically influenced by the semiconductor/dielectric interface. In order to gain a better understanding of these influences, we study the effects of different dielectric surfaces on charge transport by in-situ electrical characterisation of OTFTs during layer-by-layer growth of the OSC. This technique offers the opportunity to study the nature of the OSC/dielectric interface and the relevant OSC layers without the influence of chemical degradation by sample transfer or storage under ambient conditions. Only a few groups worldwide are following this approach [1, 3–5] and there is still ample room for improvement. Especially the nature of charge trapping and its influence on the transistor performance is not well understood up to now. In circuit design the most critical parameters of an OTFT are related to the switching behaviour; thus it is highly desirable to achieve a near-zero onset-voltage *V*_on_ and threshold-voltage *V*_th_, respectively, and a steep subthreshold slope of the drain-current, not exceeding a few hundred millivolts per decade. Those parameters, all directly related to the trap-density at the OSC-insulator-interface, are evaluated for pentacene-coverage of 1 to 22 ML on both a SiO_2_ dielectric (150 nm) and on a hybrid bilayer-dielectric comprised of a combination of 150 nm SiO_2_ and 20 nm of poly((±)endo,exobicyclo[2.2.1]hept-5-ene-2,3-dicarboxylic acid, diphenylester) (PNDPE). This polymer was recently reported in highly performing OTFTs by Petritz et al. [6] and is of technological interest in complex organic circuits due to its outstanding electrical properties and the fact that it is intrinsically photopatternable. The obtained in-situ OTFT characteristics show a reduction of interface traps of up to two orders of magnitude, an increase in mobility of one order of magnitude, and a notable reduction of the subthreshold swing on PNDPE when compared to bare SiO_2_ as gate dielectric.

## 2 Experimental

**Figure 1:**
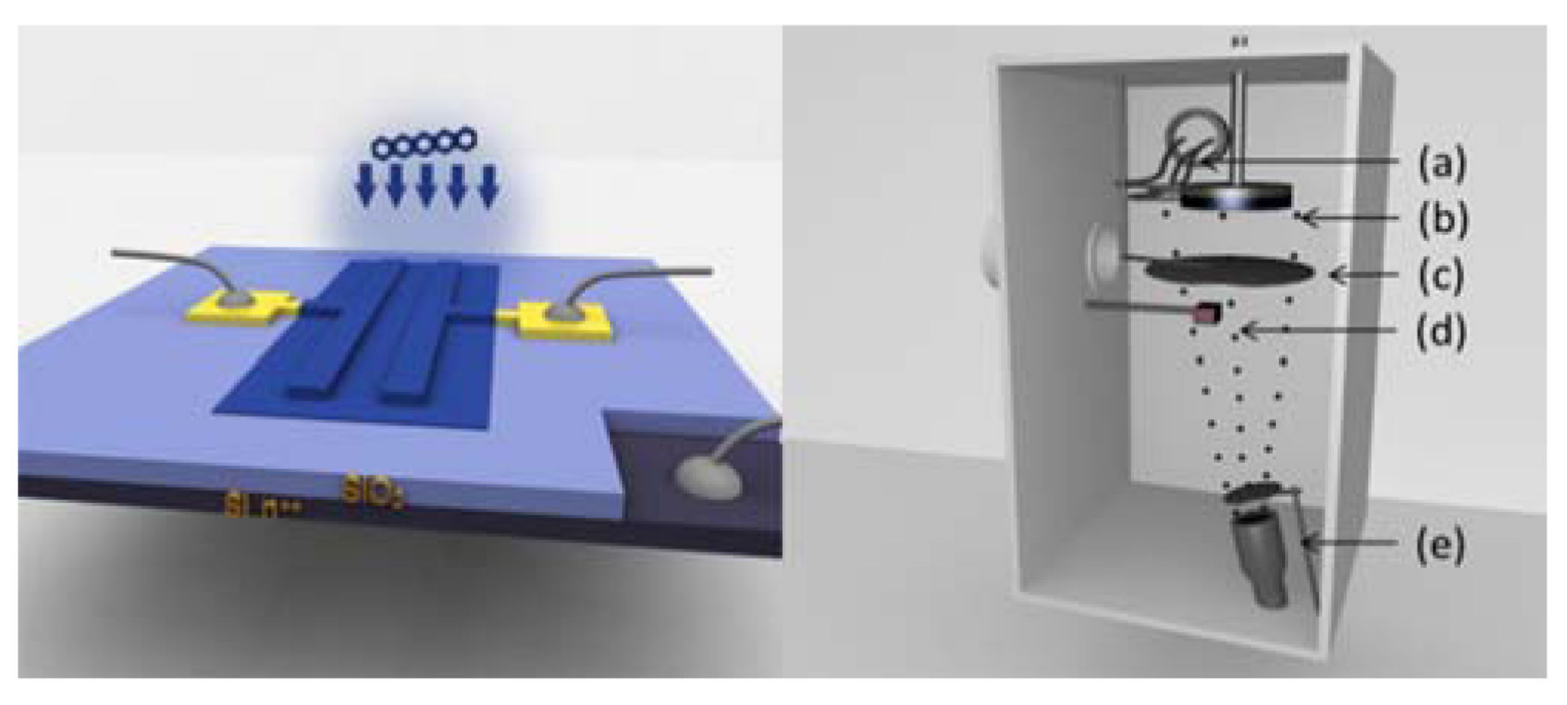
Left: Scheme of the transistors in bottom-gate/bottom-contact (coplanar) configuration used in the in-situ measurements. The wafer itself acts as gate electrode and is contacted sideways via conducting silver ink. Right: Experimental setup; (a) outside connections, (b) sample holder, (c) shutter, (d) quartz microbalance (QMB) device, (e) pentacene source.

Model transistors in bottom-gate/bottom-contact (coplanar) configuration as depicted in Fig. 1 are manufactured for the electrical in-situ characterisation. For our experiments we use p^++^ doped silicon wafer pieces from Siegert Wafer (resistivity <0.01 Ω cm, orientation ⟨100⟩) of size (10 × 10) mm^2^ with a 150 nm of (dry) thermal SiO_2_ on top as gate dielectric layer. The bulk of the wafer serves as the gate electrode. All preparation steps are performed in a cleanroom environment. The substrates are cleaned subsequently in acetone and isopropanol for 5 minutes each, assisted by sonification. Following a 30 s oxygen-plasma treatment in order to rid the substrate of organic contaminants and solvent residue, the gold source/drain contacts are evaporated through a shadow-mask at high-vacuum conditions (~10^−6^ mbar) via e-beam evaporation. Optionally, prior to the deposition of the gold electrodes, an approximately 20 nm thick layer of PNDPE (provided by University of Leoben, Chair of Chemistry of Polymeric Materials, [7]) is spin-coated (4000 rpm, 30 s) onto the SiO_2_ from a 10 mg/ml solution in Anisol (Sigma-Aldrich) and UV-cured under argon atmosphere at 254 nm (exposure dose ~1 Jcm^−2^). The finished devices with channel width *W* = 4 mm, and channel length *L* = 25 μm are mounted on a homebuilt sample-holder and the source (S)- and drain (D)-terminals are contacted via magnetically mounted contact needles. The gate is contacted sideways via a copper wire and conductive silver ink. The sample-holder along with the contacted device is installed into a high-vacuum chamber (UNIVEX 350 from Oerlikon Ley-bold Vacuum GmbH) and evacuated to a base pressure of 3 × 10^−7^ mbar. After pre-heating the pentacene (TCI, Tokyo Chemical Industries, Co. Ltd., purified twice by train sublimation) in a molecular beam epitaxy cell (Creaphys) for about half an hour, the OSC is evaporated onto the electrode-structures ML by ML at a low deposition rate of approximately 1 Å/min. The OSC-coverage is monitored with a quartz crystal microbalance and cross-checked by ex-situ atomic force microscopy (AFM, Dimension 3100 from Veeco Instruments). After completion of each ML, transfer curves are recorded via electrical feedthroughs and an externally connected parameter analyser (mb-Technologies). The measurements are performed on bare SiO_2_, and on a combination of SiO_2_ and PNDPE as gate dielectric layer. The final OSC-thickness is ~22 ML, corresponding to a nominal coverage of 35 nm of pentacene.

## 3 Results and discussion

### 3.1 Pentacene growth

AFM measurements on different samples with coverage reaching from submonolayer up to a few monolayers of pentacene reveal a layer-plus-island growth mode (Stranski–Krastanov) on both SiO_2_ and PNDPE as growth matrix. The first layer created is usually a closed wetting layer of standing up molecules [8], on top of which islands of standing molecules start to form as more material is deposited. The orientation of the deposited molecules is indicated by the step-height between terraces and is determined by AFM to be 1.6 ± 0.1 nm which corresponds to the Bravais vector ***c*** of the pentacene molecule as determined by Campbell et al. [9].

### 3.2 Layer by layer characterisation

**Figure 2:**
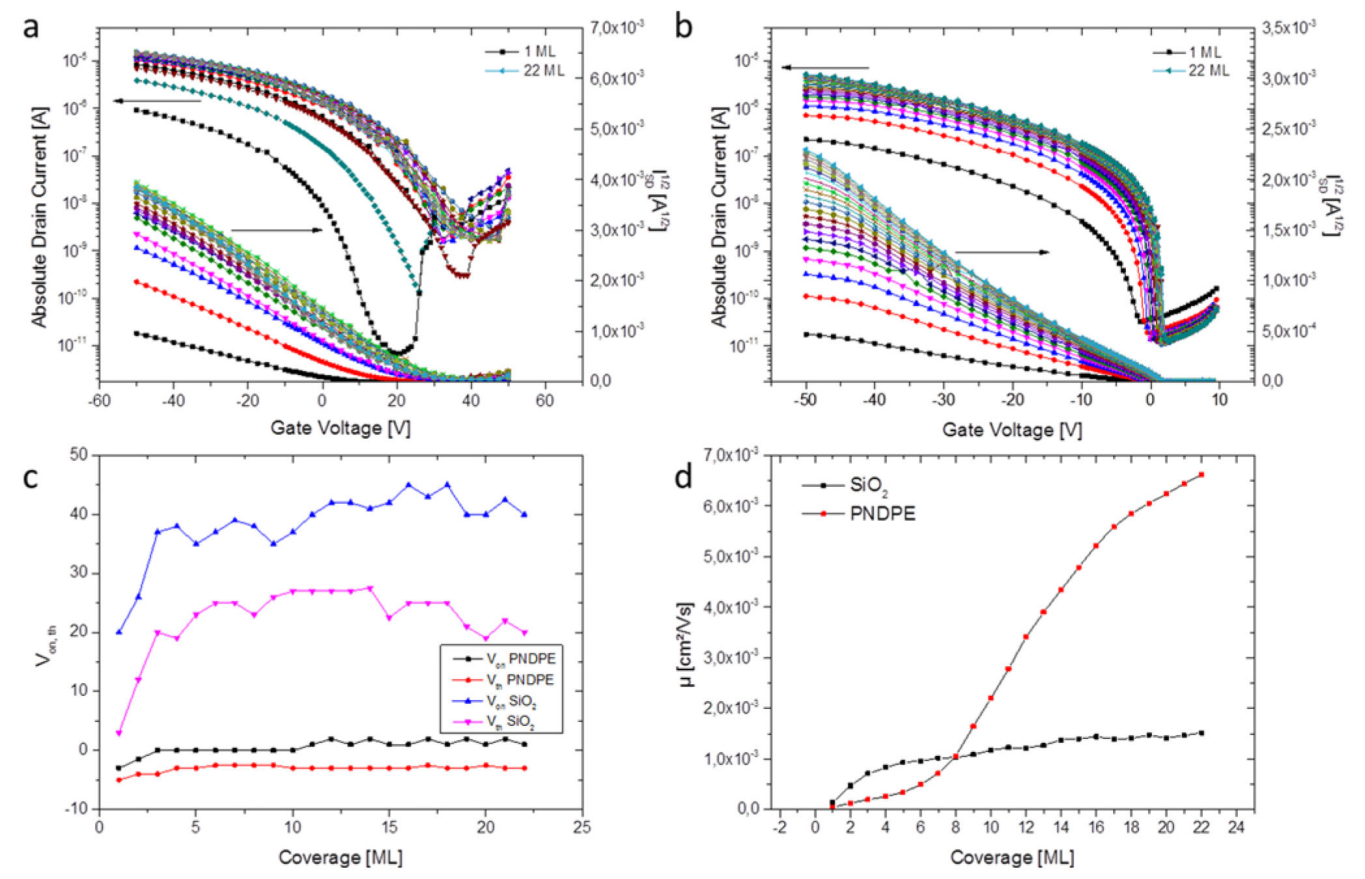
Top row: Transfer curves in forward direction for coverage 1 to 22 ML at *V*_DS_ = −70 V. a) Measurement series on SiO_2_ as gate dielectric layer. A strong shift of the onset- and threshold-voltage is visible, along with a gradual reduction in the on/off-ratio of the device. b) Measurement series on PNDPE as gate dielectric layer. The onset- and threshold-voltages are very stable compared to measurements on SiO_2_ as dielectric layer, only a slight shift in the range of ~2 V is observed. The on/off-ratio is stable and greatly improved for all coverages and the subthreshold-slope of the device is remarkably steep. c) Evolution of threshold- and onset-voltage for SiO_2_ and PNDPE as gate dielectric layer. d) Typical evolution of the mobility on SiO_2_ and PNDPE.

Transfer curves are recorded in-situ during ML by ML OSC growth as schematically shown in Fig. 1. Figure 2a depicts a complete series of transfer curves for a range of coverage from 1 to 22 ML for a bottom-gate/bottom-contact device with a 150 nm of SiO_2_ as gate dielectric layer. The curves are recorded at *V*_DS_ = −70 V. At a coverage of 1 ML the device turns on at about *V*_on_ = +20 V. For the subsequent layers the onset voltage shifts towards more positive voltages with each additional pentacene layer. The total shift in onset voltage is about Δ*V*_on_ = +20 V. This behaviour is observed in all devices with SiO_2_ as gate dielectric layer and is, at least qualitatively, reproducible. There is, however, quite a strong variation in the starting value, i.e. the onset-voltage *V*_on_ for the first ML may vary over tens of volts. This variation in the onset-voltage is strongly correlated to the density of OH-groups at the OSC/dielectric-interface as described in more detail in Refs. [10, 11]. OH-groups act as electron traps, which have a great influence on the switching on behaviour of an OTFT, shifting *V*_on_ in the positive direction. The surface area density of OH-groups on a SiO_2_-dielectric is difficult to control, highly sensitive to various surface treatments [5, 12], and thus also strongly depends on the humidity during sample preparation_,_ the timespan between preparation steps, etc. Starting from the individual *V*_on_ for each device, the evolution of *V*_on_ with coverage is, however, very similar for all devices with SiO_2_ as gate dielectric, corresponding to a voltage shift of Δ*V*_on,sat_ ~ 15–20 V. A similar shift with similar saturation value Δ*V*_th,sat_ ~ 20 V is observed for the threshold voltage in devices with pure SiO_2_ dielectric (compare *V*_on_ SiO_2_ and *V*_th_ SiO_2_ in Fig. 2c). According to Fig. 2c, all voltage shift effects saturate at a coverage of approximately 4 to 6 MLs.

Liu et al. [3] reported a different behaviour of the threshold-voltage dependency on the coverage, namely a drop-off of *V*_th_ around 3 ML, which is not observed in our experiments, wherein the threshold-voltage is increasing steadily with increasing coverage up to a saturation point, similar as reported by Fiebig et al. [5]. The latter also observed a large sample-to-sample variation in the mobility saturation coverage ranging from 4 ML to over 20 ML. The higher the absolute mobility values, the lower the coverage value at which the mobility saturates (mobility saturation coverage). These variations indicate a dominant influence of extrinsic factors such as the contacts and the nature and condition of the dielectric surface (OH-groups, roughness, humidity, etc.) both determining the semiconductor morphology. Moreover, the OSC morphology is well known to depend on the evaporation rate [11]; in Ref. [1] also a dependence of the saturation thickness on the OSC-evaporation rate is reported. In our experiments the main focus is set on the nature of the OSC/dielectric interface, therefore all deposition parameters are kept constant.

The voltage shifts as a function of coverage can be explained by the gradual filling of deep electron trap states at or near the interface upon OSC evaporation resulting in the formation of fixed negative charges. As the pentacene film grows thicker, more and more trap states are filled by electrons, negatively charging the semiconductor film, and therefore requiring compensation by a positive gate bias. However, the density of negatively charged trap states decreases with increasing distance from the interface (within the Debye length) thus finally inducing a saturation of the shift. This is in good agreement with theoretical considerations by e.g. Bolognesi et al. [11], Scheinert et al. [13], Sirringhaus [14] and Fiebig et al. [5]. Those electron traps could arise from grain boundaries, intrinsic impurities in the pentacene powder or impurities caused by chemical reaction with oxygen or water molecules in the residual gas [5].

Figure 2b depicts the same series of measurements for a device with a combination of 150 nm SiO_2_ and 20 nm of PNDPE as dielectric layer. Δ*V*_on_ and Δ*V*_th_ are considerably smaller, the curves are almost congruent, and the device turns on close to 0 V for all coverages, which indicates that the density of interface trap states is strongly reduced on PNDPE when compared to bare SiO_2_. This is consistent with previous observations by Petritz et al. [6]. There is still a small visible shift in onset-voltage; it is, however, reduced by approximately one order of magnitude when compared to the pure SiO_2_ dielectric.

The strong reduction of interface traps is also evident when examining the subthreshold-behaviour of the transfer curves depicted in Fig. 2a and b, with SiO_2_ and PNDPE respectively, as gate dielectric layers. The subthreshold swing is a measure for the density of shallow charge carrier traps at the interface [6]. The density of interface trap states *N*_SS,max_ of the manufactured devices can be estimated employing the method reported by Rolland et al. [15]

(1) $$N_{\mathrm{SS},\max}\;=\left[\frac{q\;\log\;(e)\;S}{\mathrm{kT}}-1\right]\frac{C_{i}}{q^{2}},$$

where *C_i_* is the gate dielectric capacitance, *q* the elementary charge, *k* the Boltzmann constant, and *T* the temperature. The calculated values for the interface trap density are listed in Table 1.

The interface trap density is reduced by up to two orders of magnitude on PNDPE-modified SiO_2_. Devices with PNDPE as dielectric layer show almost ideal switch-on behaviour, demonstrated by a subthreshold swing as low as 300 mV/dec, and no hysteresis (see supplementary information). The lowest subthreshold swing in our experiments achieved on SiO_2_ is as high as 3 V/dec. The on/off-ratio of the source-drain currents on SiO_2_ is decreasing with increasing coverage, whereas it is not only larger in general on the PNDPE-modified interface, but also increases there with higher coverage. On/off-ratios as high as 10^8^ are achieved on PNDPE-modified SiO_2_.

### 3.3 Mobility

**Figure 3:**
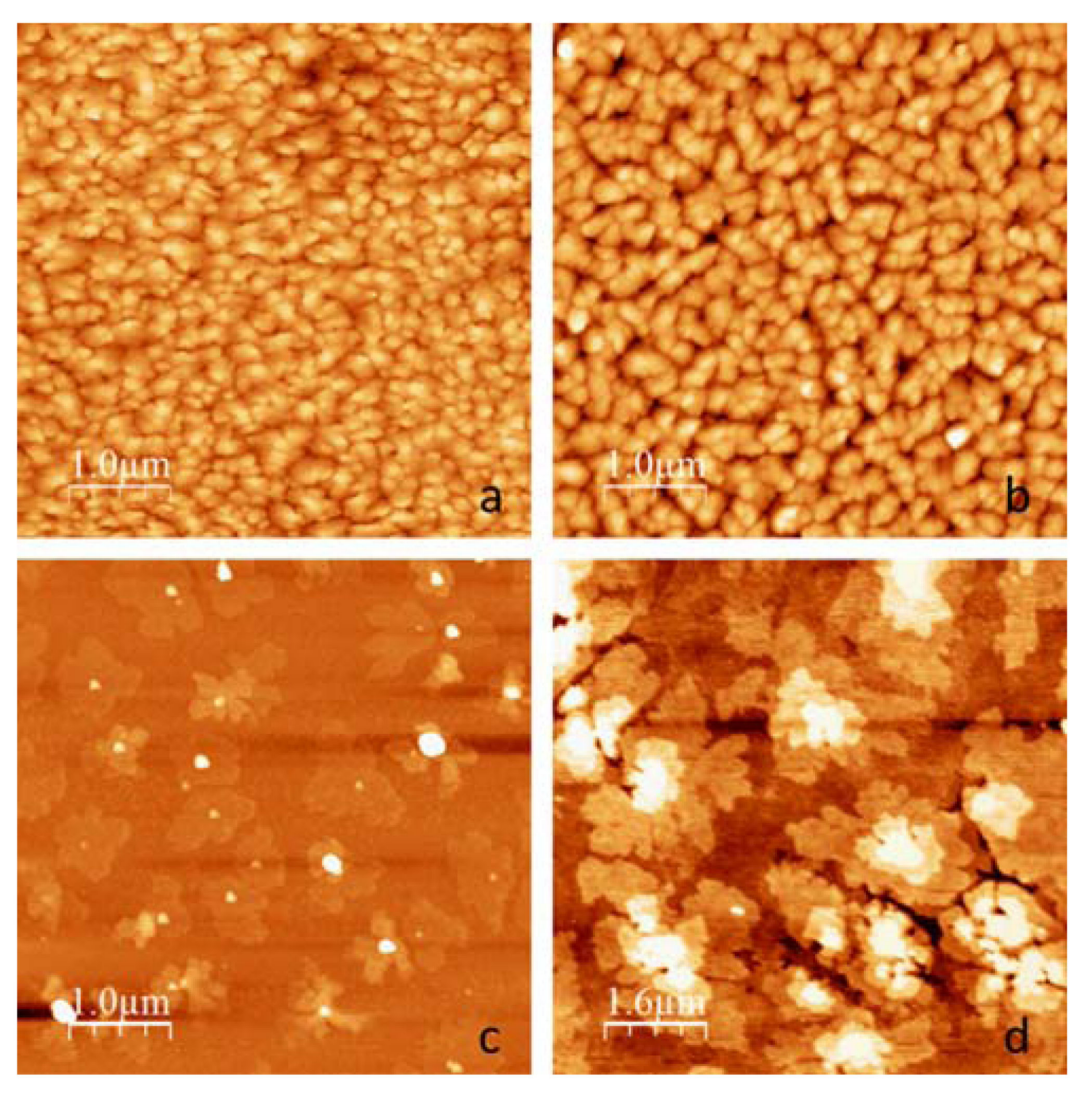
a) Pentacene growth on PNDPE, *d* = 35 nm; b) pentacene growth on SiO_2_, *d* = 35 nm. c) Approx. 0.5 ML of pentacene on SiO_2_, pronounced 3D-growth features appear before the first ML closes. d) Approx. 3 ML of pentacene on PNDPE: again 3D-growth features are visible.

As previously shown by Lassnig et al. [16], first percolation paths within the transistor channel are formed at a coverage of approximately 0.7 ML allowing for a current to flow in the yet incomplete first ML. The electrical submonolayer characteristics are discussed in detail in a recent publication by Cramer et al. [17]. With increasing OSC-coverage the number of available charge carriers and thus the mobility μ increases significantly up to coverage of approximately 4 ML, see Fig. 2d. While most groups report a saturation thickness in the range of 3 to 7 ML for mobility and SD-currents [1, 3], we do not observe a saturation of those two parameters in our experiments. We observe – on both dielectric interfaces – a continuous yet unsteady increase of mobility and SD-currents up to a coverage of 22 ML (Fig. 2d), which rather agrees with the observations of Fiebig et al. [5] who found a similar behaviour. The origin of this effect is to this point unknown and could be related either to 2D versus 3D growth effects [1] or to coverage related contact effects at the channel edges, where OSC and SD-electrodes meet [16]. The morphology of 22 ML (~35 nm) of pentacene on both SiO_2_ and PNDPE is depicted in Fig. 3. The layer in both cases exhibits grain sizes smaller than 0.5 μm and, generally, a comparable growth mode with a more pronounced 3D-growth on SiO_2_ compared to PNDPE. This is a reasonable result when comparing the mobility values for the presented devices, which are of the same order of magnitude, and can also explain the smaller slope of the evolution of SD-currents on SiO_2_, Fig. 3b; the undermost layers are not completed as effectively on SiO_2_ as on PNDPE. The semiconductor morphology or more specifically the gradual completion of the undermost MLs can explain the continuous increase in mobility and SD-currents; each deposited nominal ML adds to the 3D-growth features of the OSC layer while simultaneously gradually filling and completing underlying layers [1]. The continuous increase in mobility can also be explained by a gradual decrease of the access resistance with increasing coverage [16]; the contact between electrodes and OSC is steadily improved as OSC material is deposited and thus lowering the access resistance. The absolute mobility values are comparable to bottom-gate/bottom-contact devices without electrode treatment reported in literature. On PNDPE the obtained mobility is approximately three times larger than on pure SiO_2_ which we attribute to a more strongly pronounced 3D-growth on SiO_2_ and the significantly reduced Coulomb scattering of charge carriers due to the reduction of interface trap density [18].

## 4 Summary and conclusions

We have presented in this contribution an electrical in-situ characterisation of pentacene-based organic thin film transistors in a bottom-gate/bottom-contact (coplanar) configuration with SiO_2_ only, and a combination of SiO_2_ and PNDPE as gate dielectric layer. The experimental setup allows for the determination of critical transistor parameters, layer-by-layer, under exclusion of detrimental influences like humidity or oxygen and is a versatile tool to characterise different dielectrics or OSCs. We have thusly demonstrated a method for a beneficial modification of SiO_2_ by coating it with an ultrathin additional layer of the intrinsically photo-patternable polymer dielectric PNDPE. This way we were able to manufacture devices with a subthreshold swing as low as 0.3 V/dec employing PNDPE as a surface modification and stabilization layer for SiO_2_. As demonstrated by the in-situ recording of transfer curves for a range of coverage between 1 and 22 ML, this surface-modification allows for a reduction of interface traps *N*_SS_ of up to two orders of magnitude, resulting in drastically reduced subthreshold swing and significantly improved switch-on behaviour. The off-currents of the devices with PNDPE are about two orders of magnitude lower and hence show a significantly increased on/off-ratio which may be as large as 10^8^ in top-performing devices.

## Supplementary Material

Supplementary Information 1

Supplementary Information 2

## Figures and Tables

**Table 1:** Comparison of top performing devices with either SiO_2_, or SiO_2_ and PNDPE as dielectric layer.

dielectric	SiO_2_	SiO_2_ & PNDPE
*C_i_* [nF cm^−2^]	23	19
*μ* [cm^2^ V^−1^ s^−1^]	1.6 × 10^−3^	7 × 10^−3^
*S* [Vdec^−1^]	8	0.3
*I*_on_/*I*_off_	10^4^	10^8^
Δ*U*_th_ [V]^1^	18	2.5
Δ*U*_on_ [V]^2^	15	4
*N*_SS_ [cm^−2^ eV^−1^]	1.25 × 10^13^	1.4 × 10^11^

1, 2 Absolute shift in threshold- and onset-voltage, respectively, for 1 and 22 ML.
