# Supplementary figures and images for: Electrical in-situ characterisation of interface stabilised organic thin-film transistors
Source: Phys Status Solidi Rapid Res Lett. Author manuscript; Available in PMC 2015 Oct 9. (PMC4599138; doi:10.1002/pssr.201510169)

# Pentacene oTFT with SiO<sub>2</sub> dielectric

Layer by Layer Transfer  
Characteristics

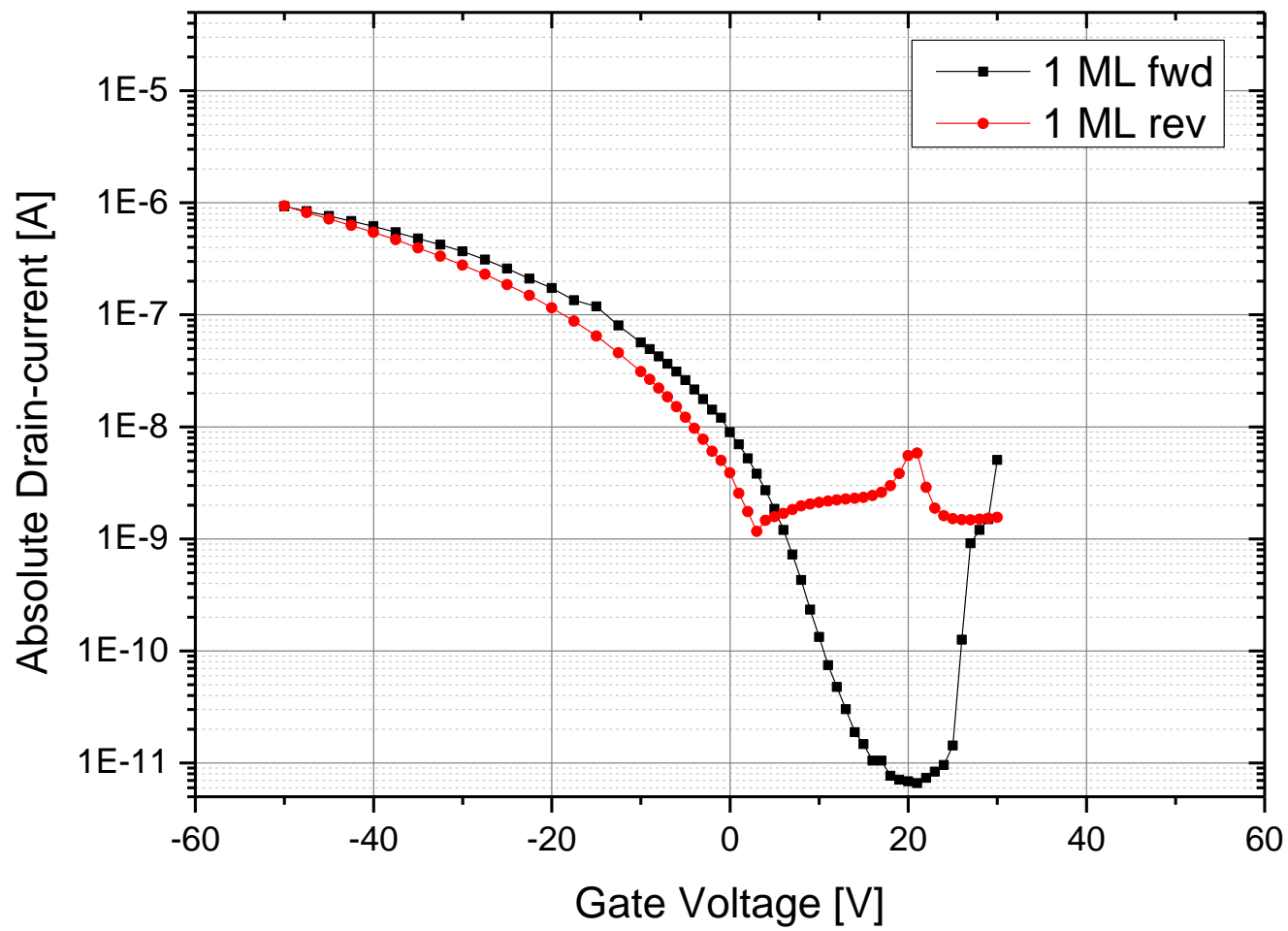

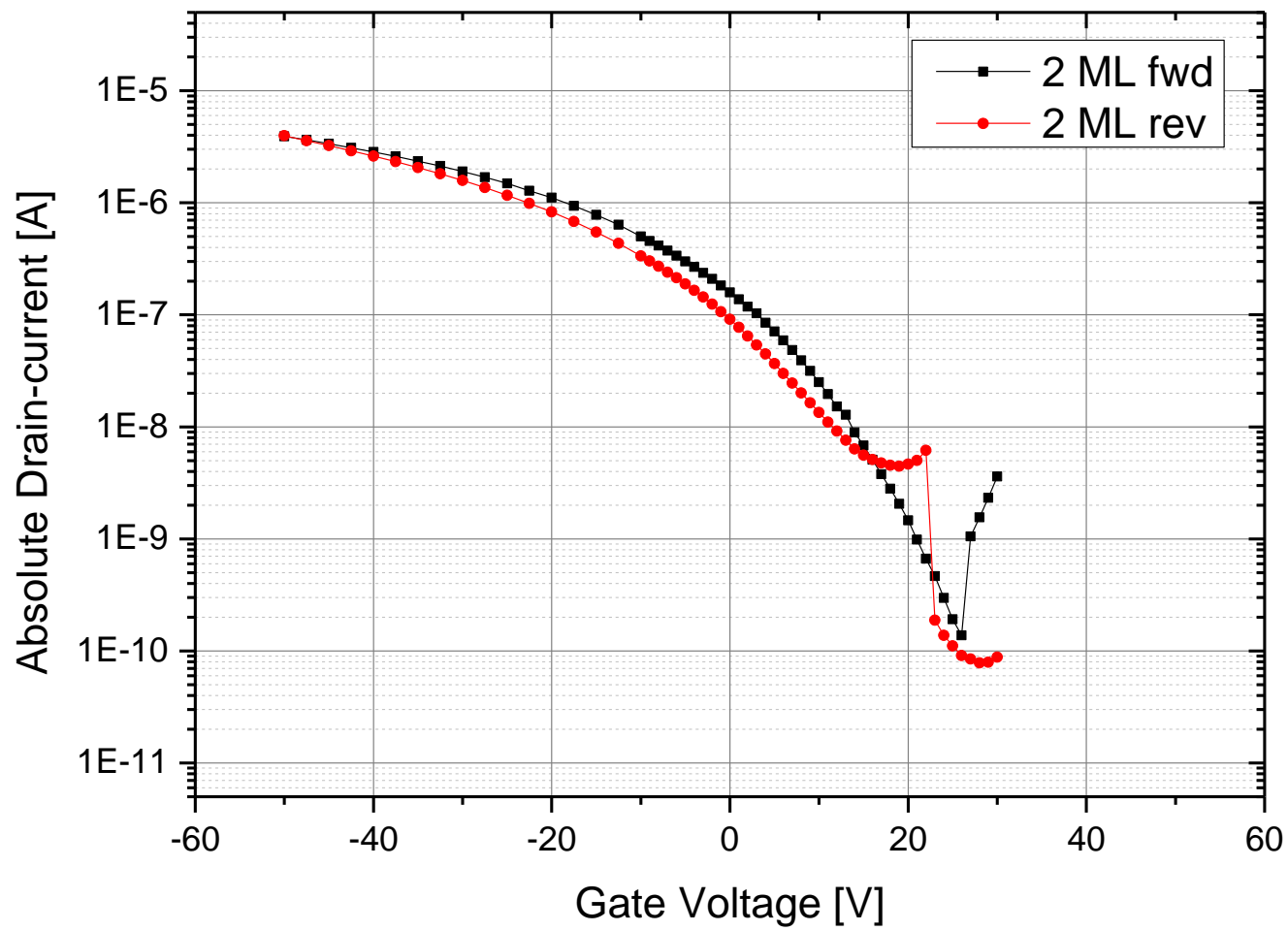

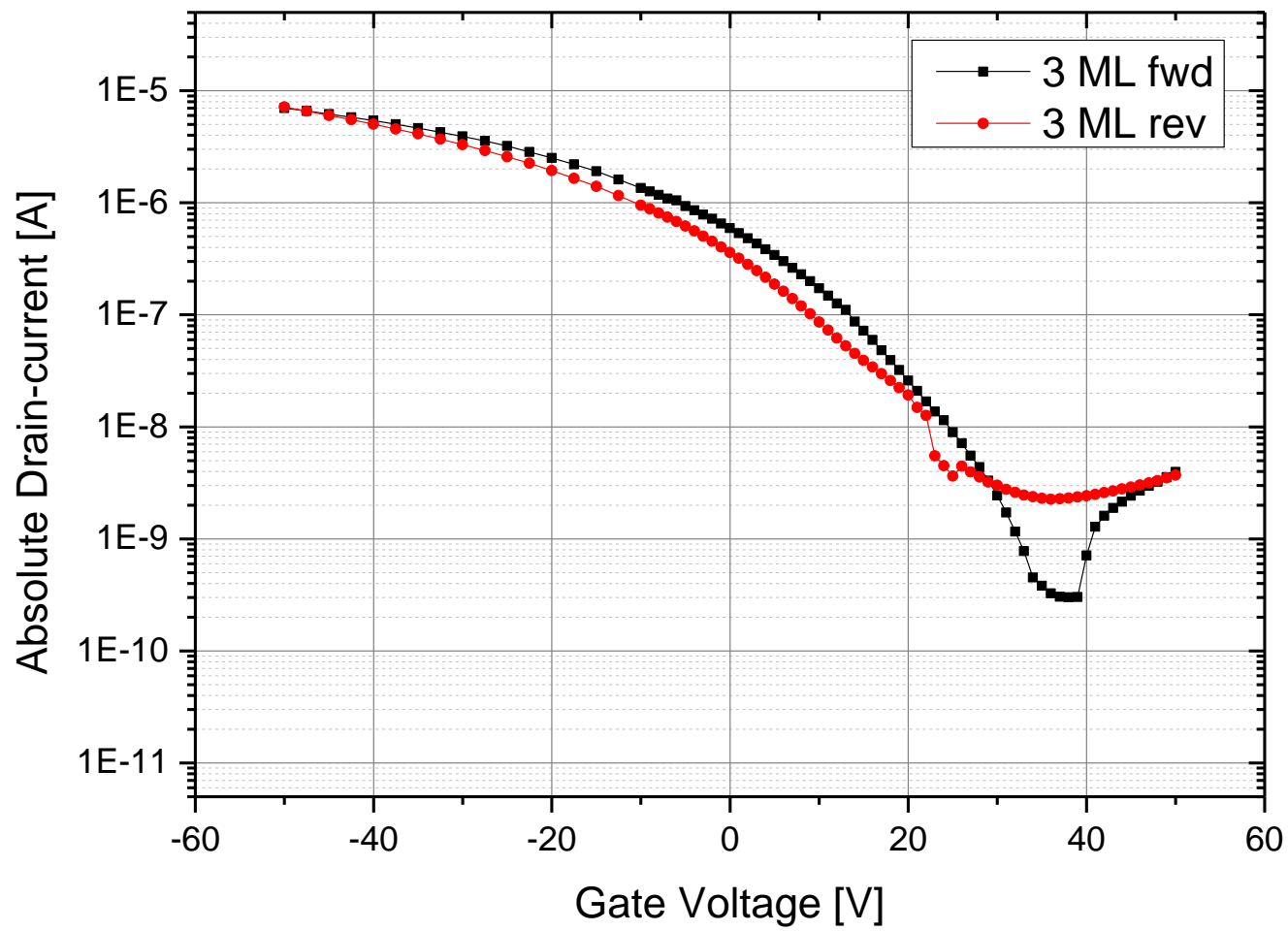

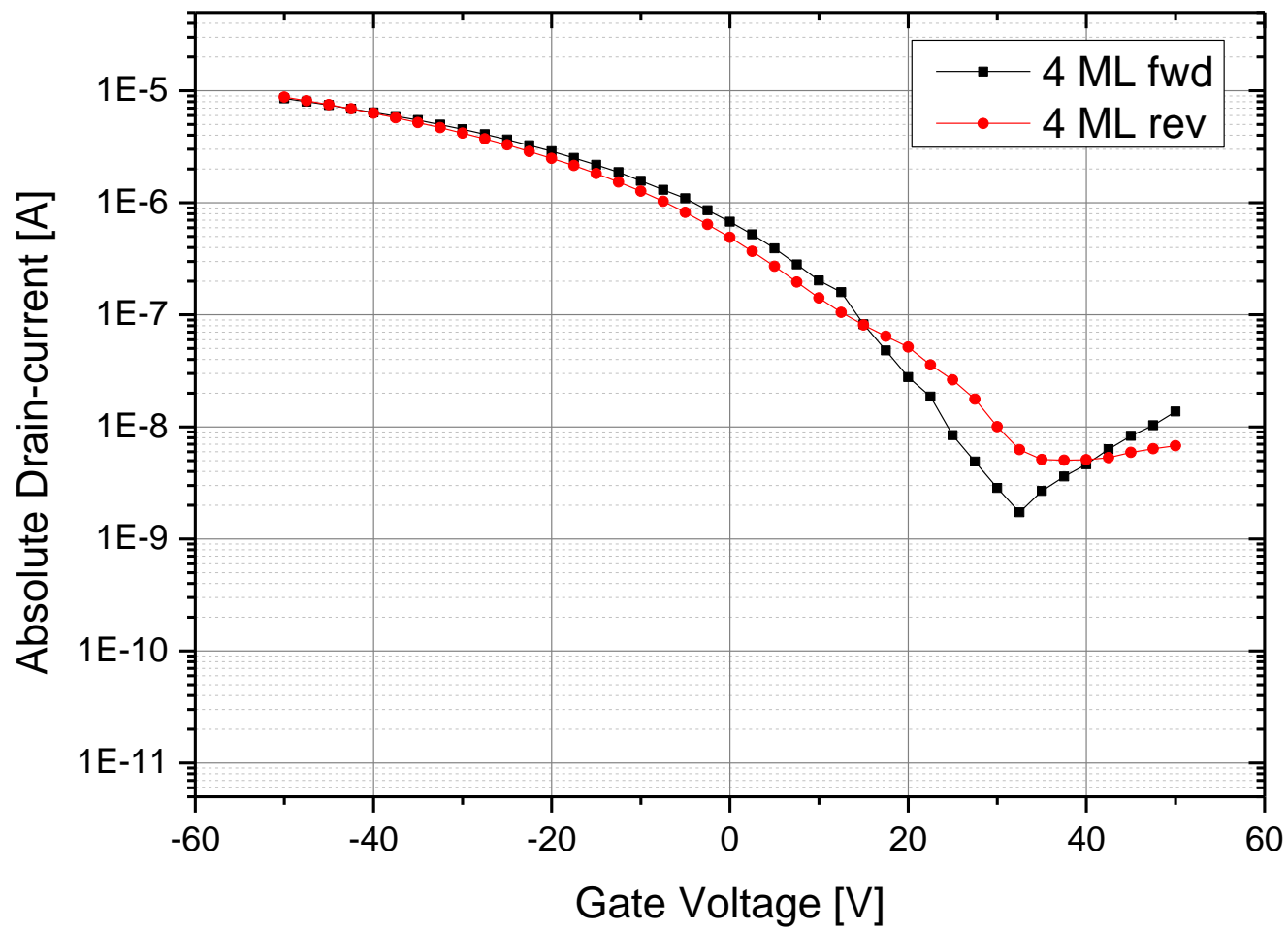

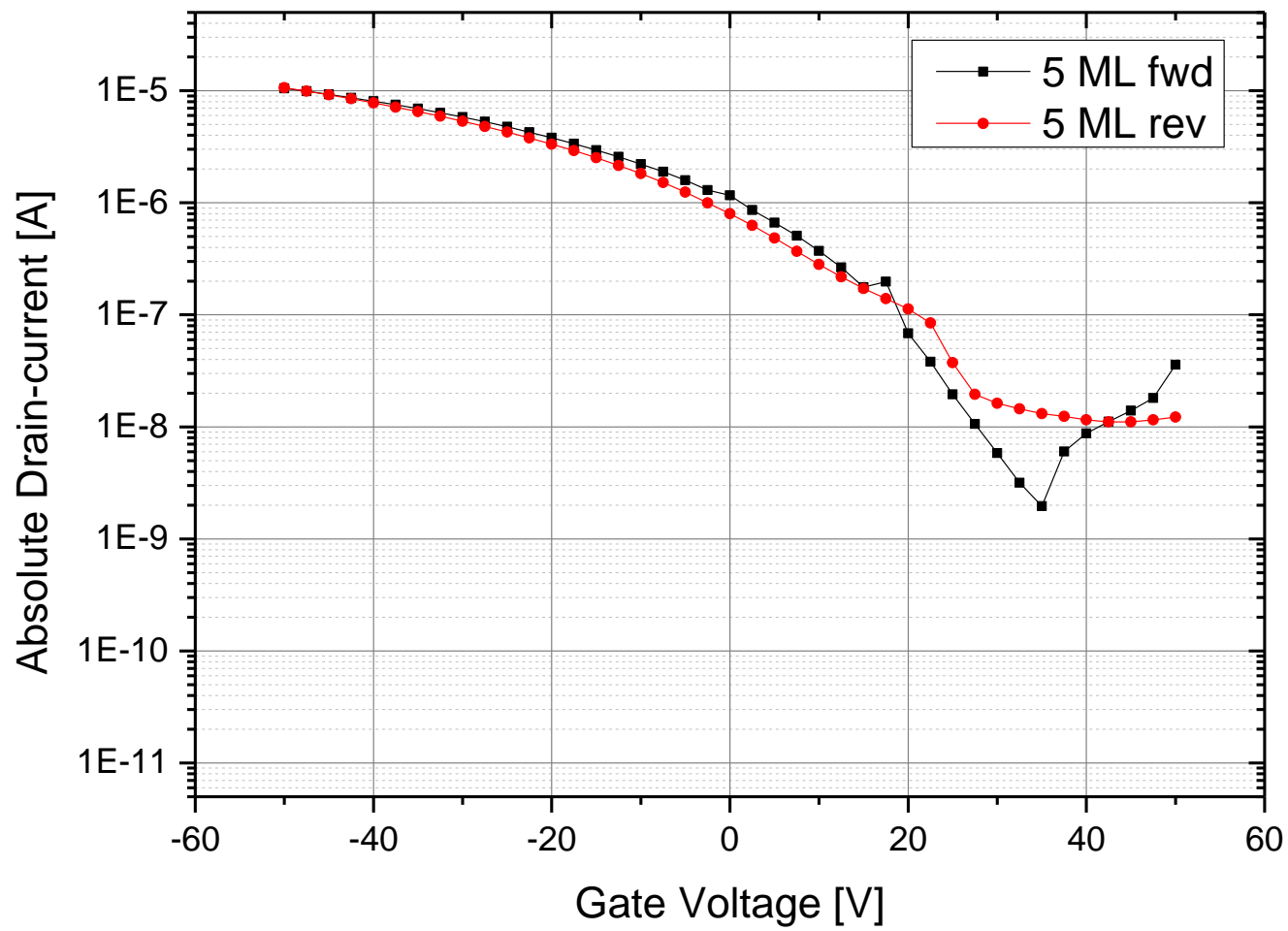

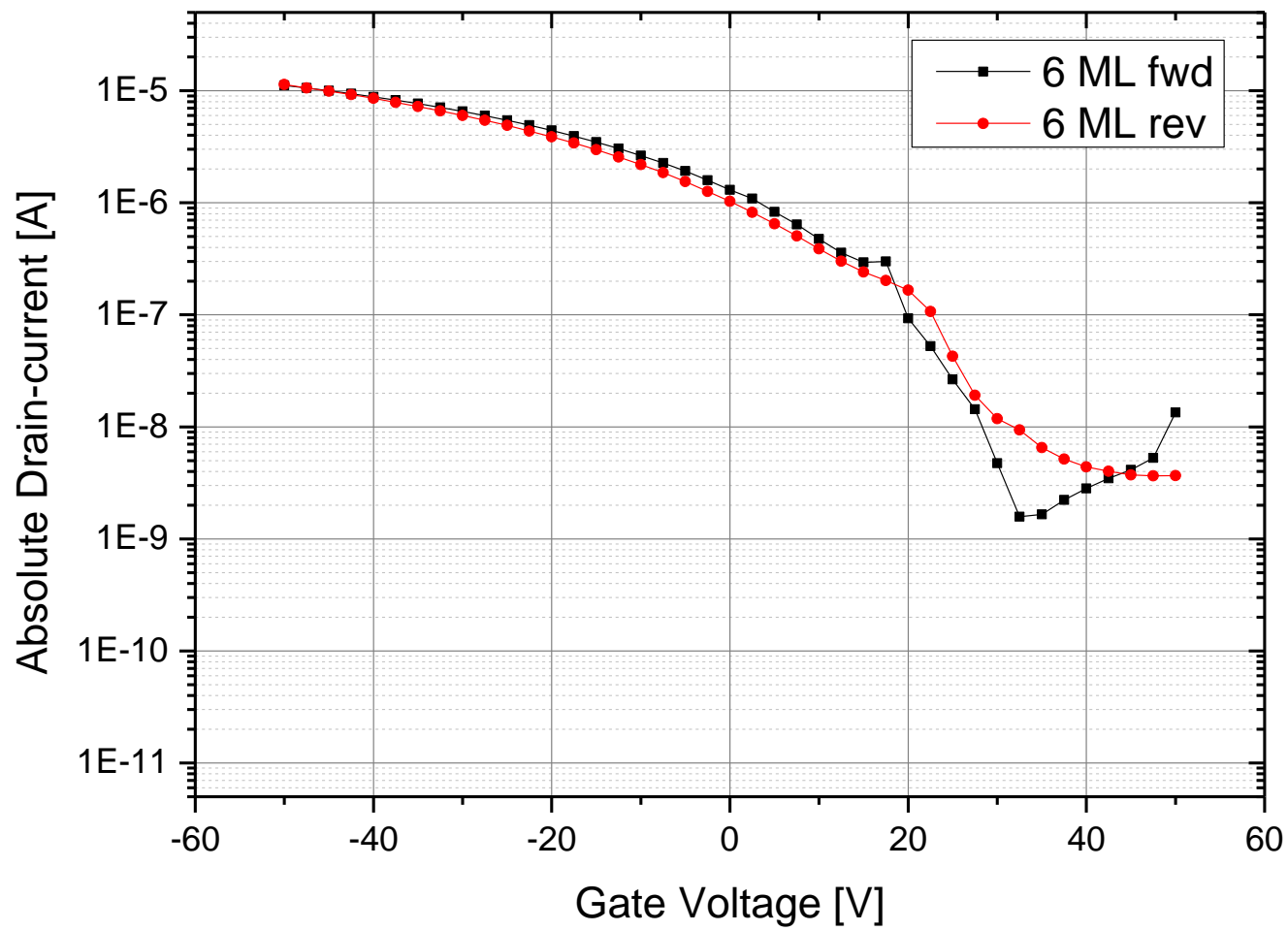

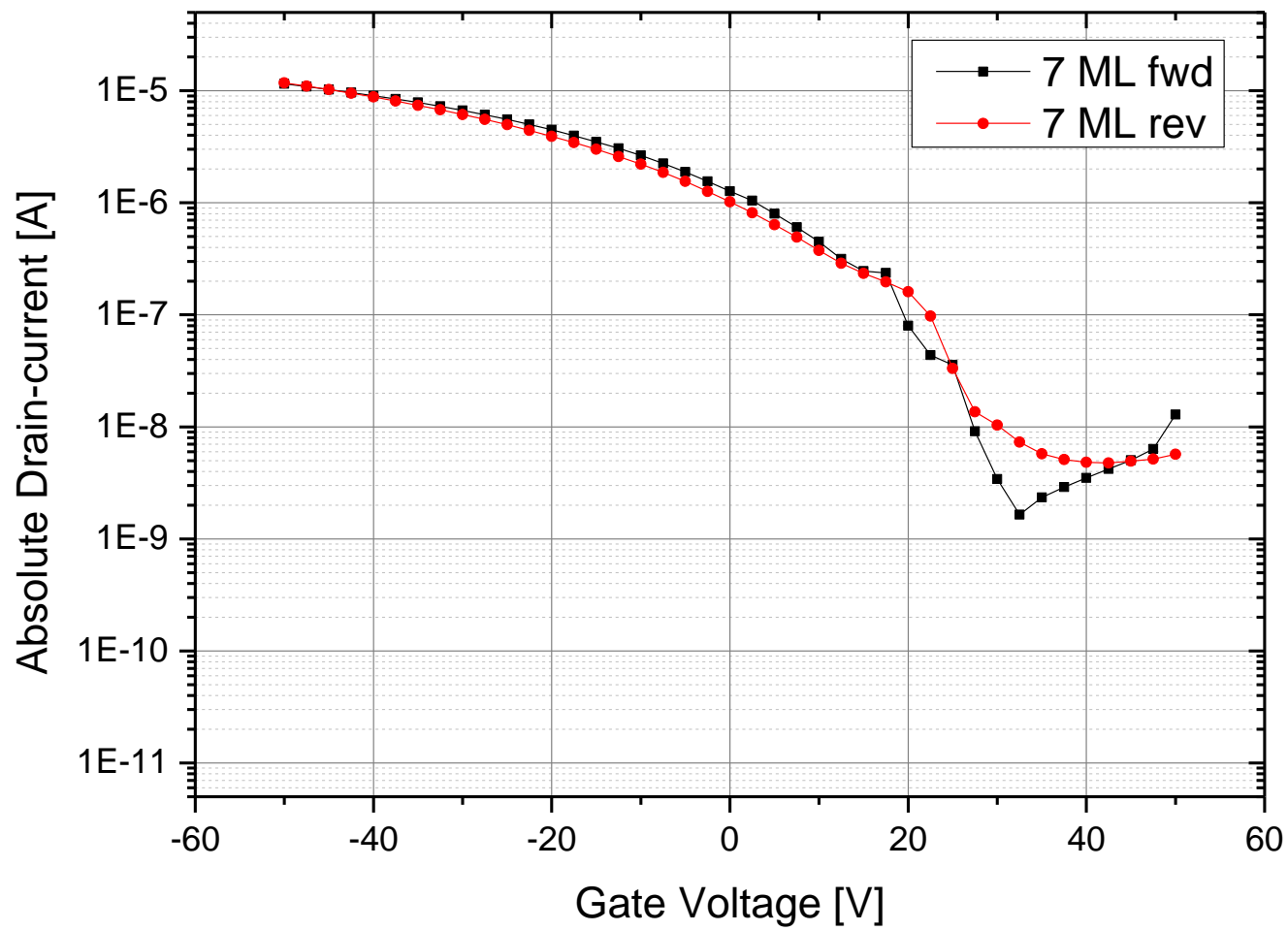

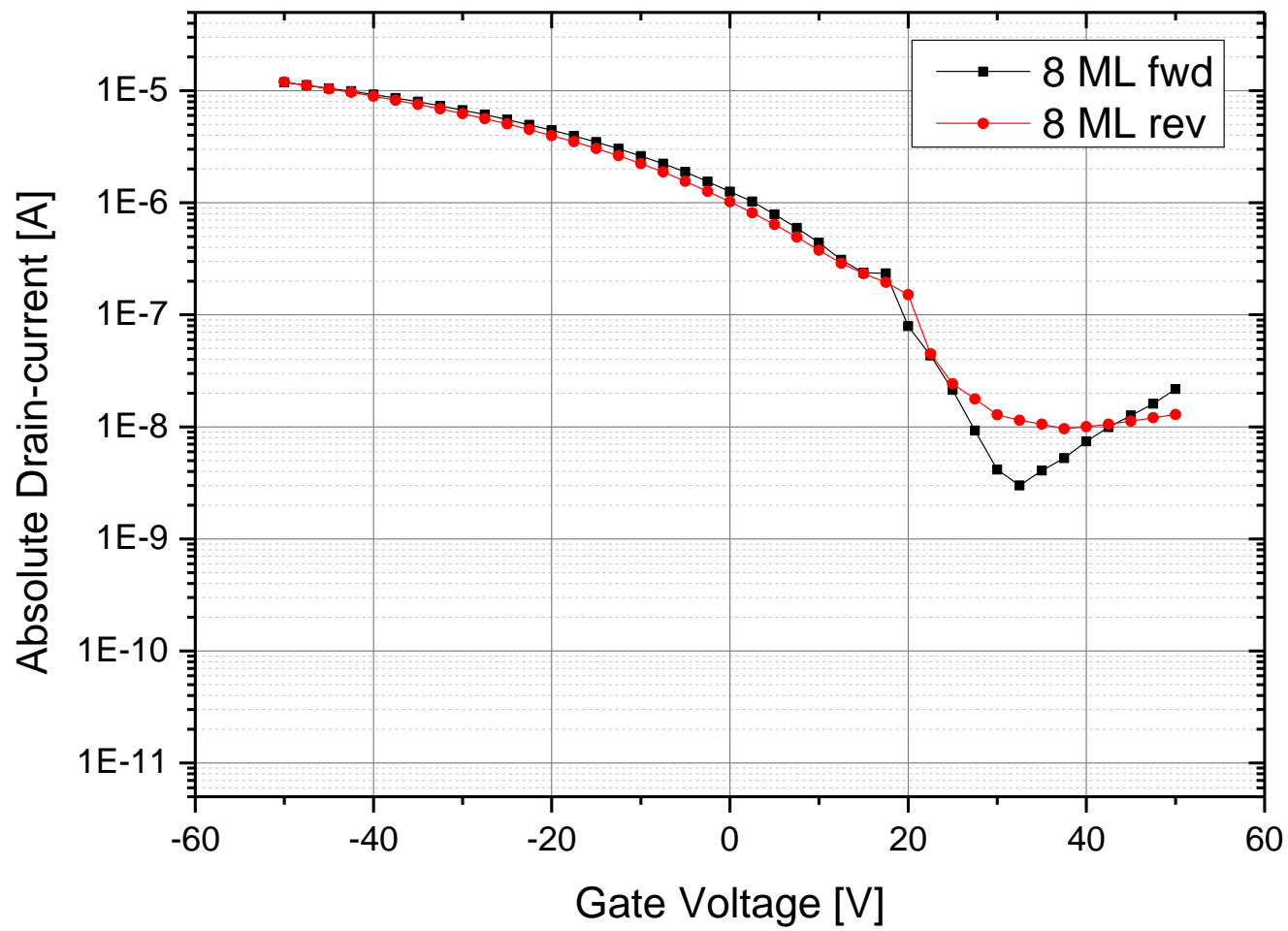

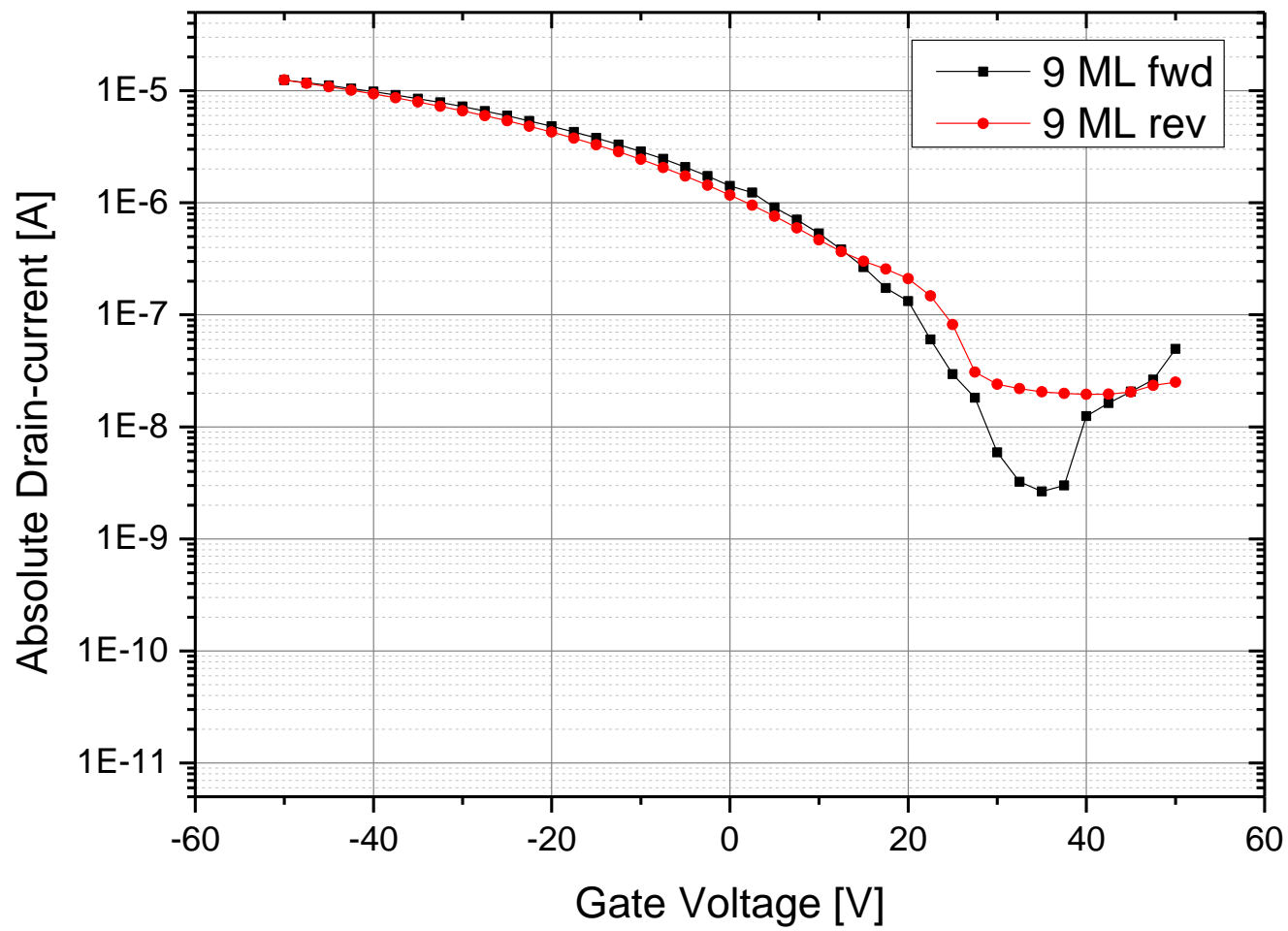

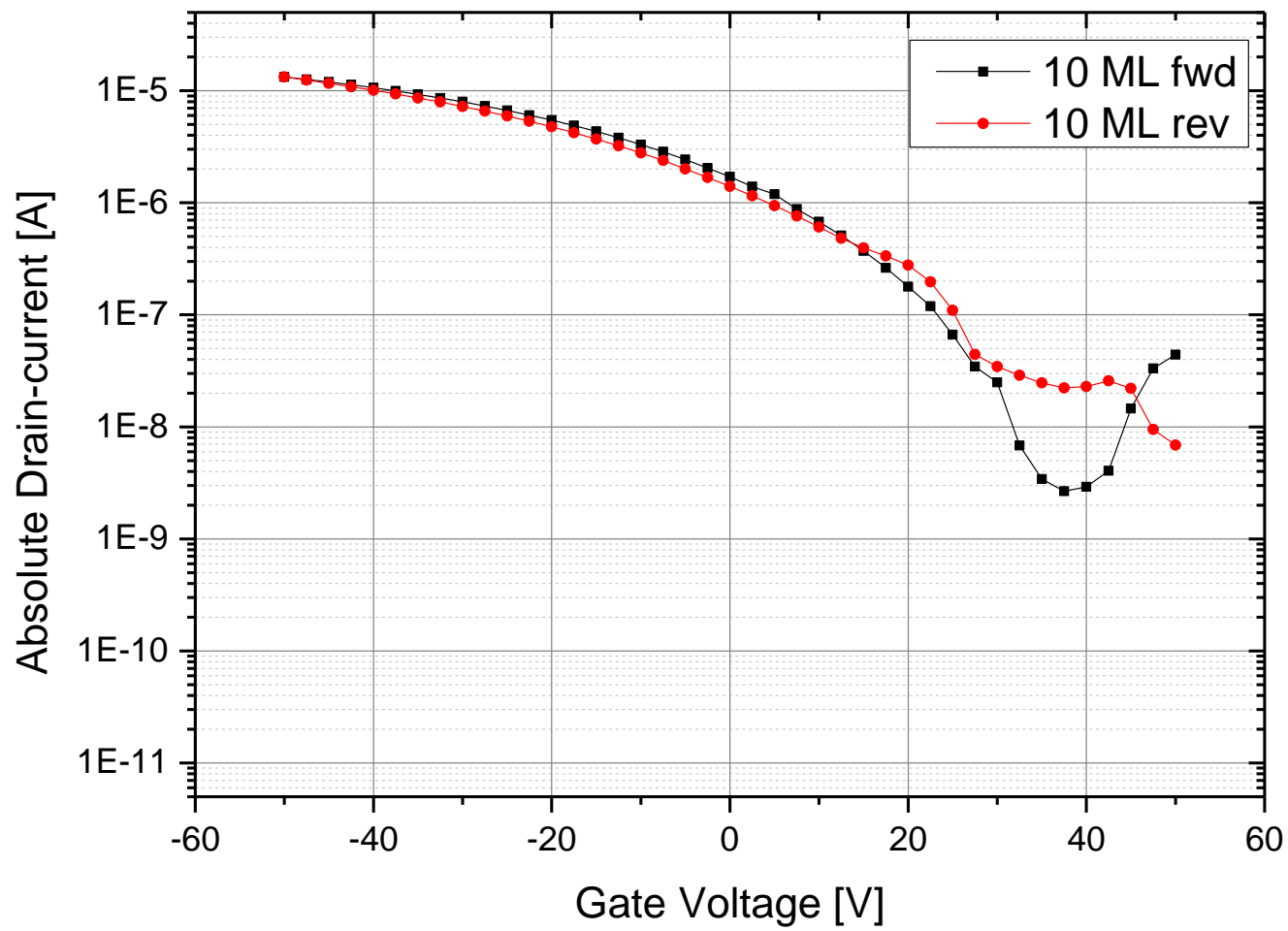

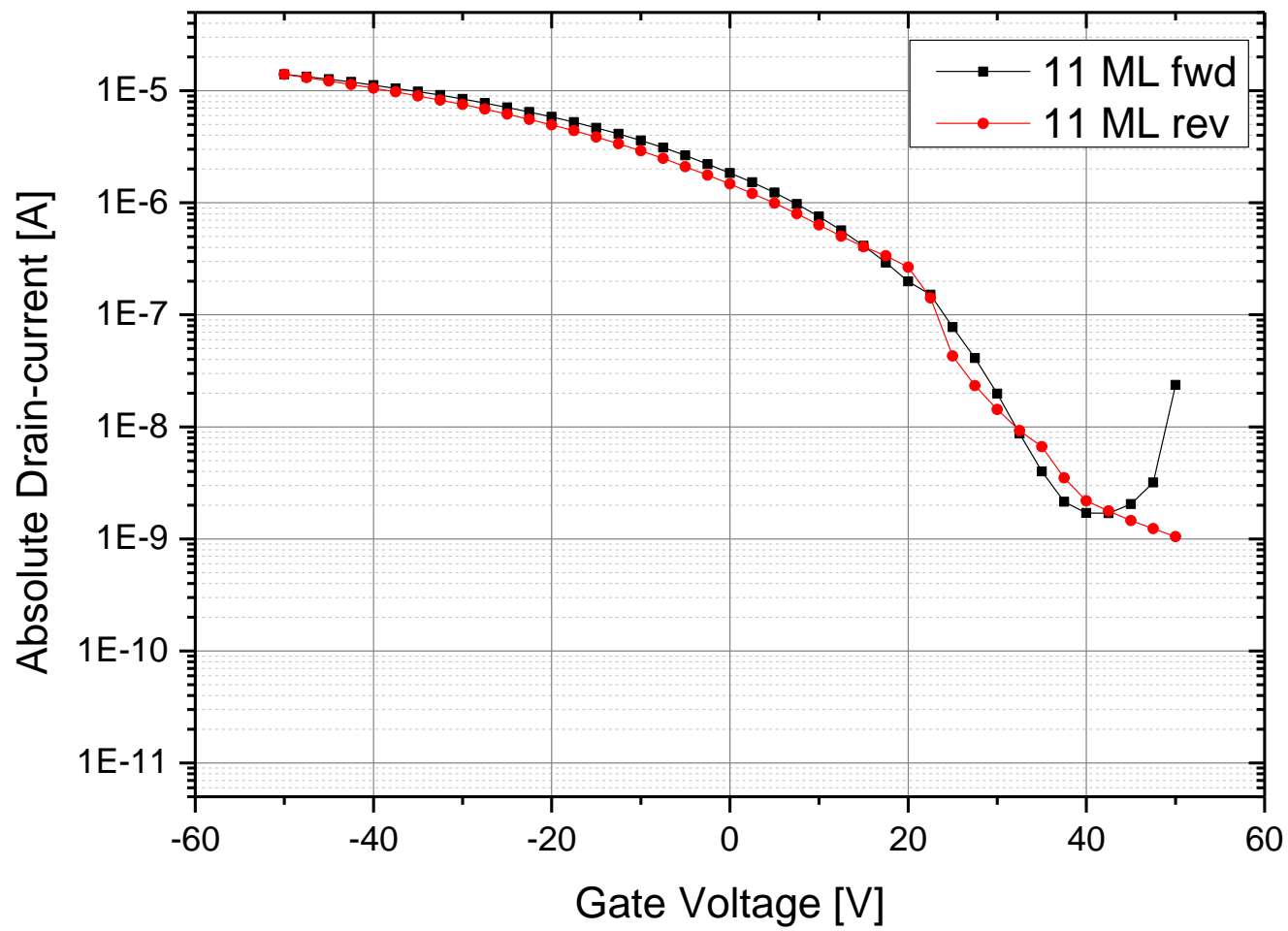

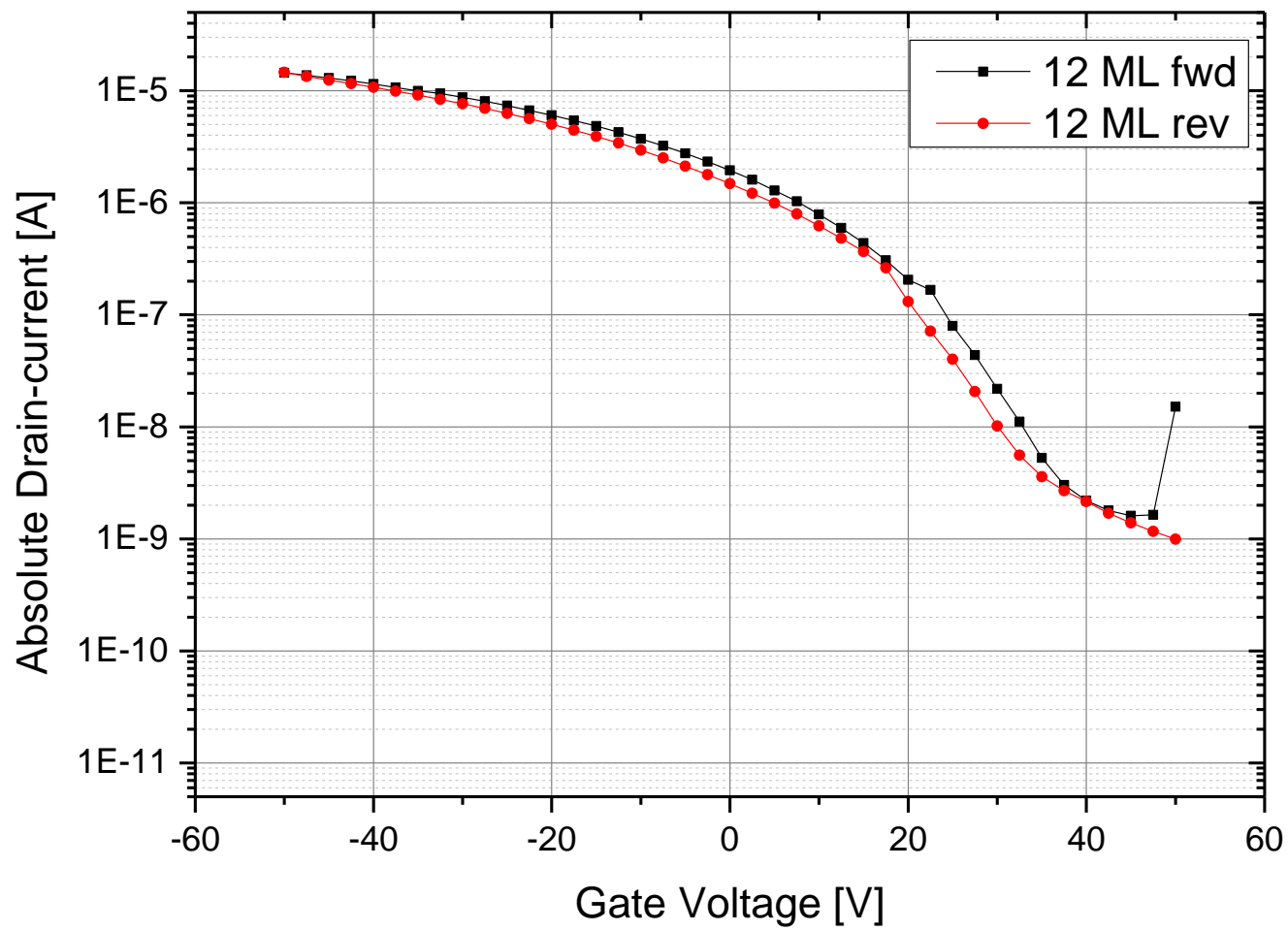

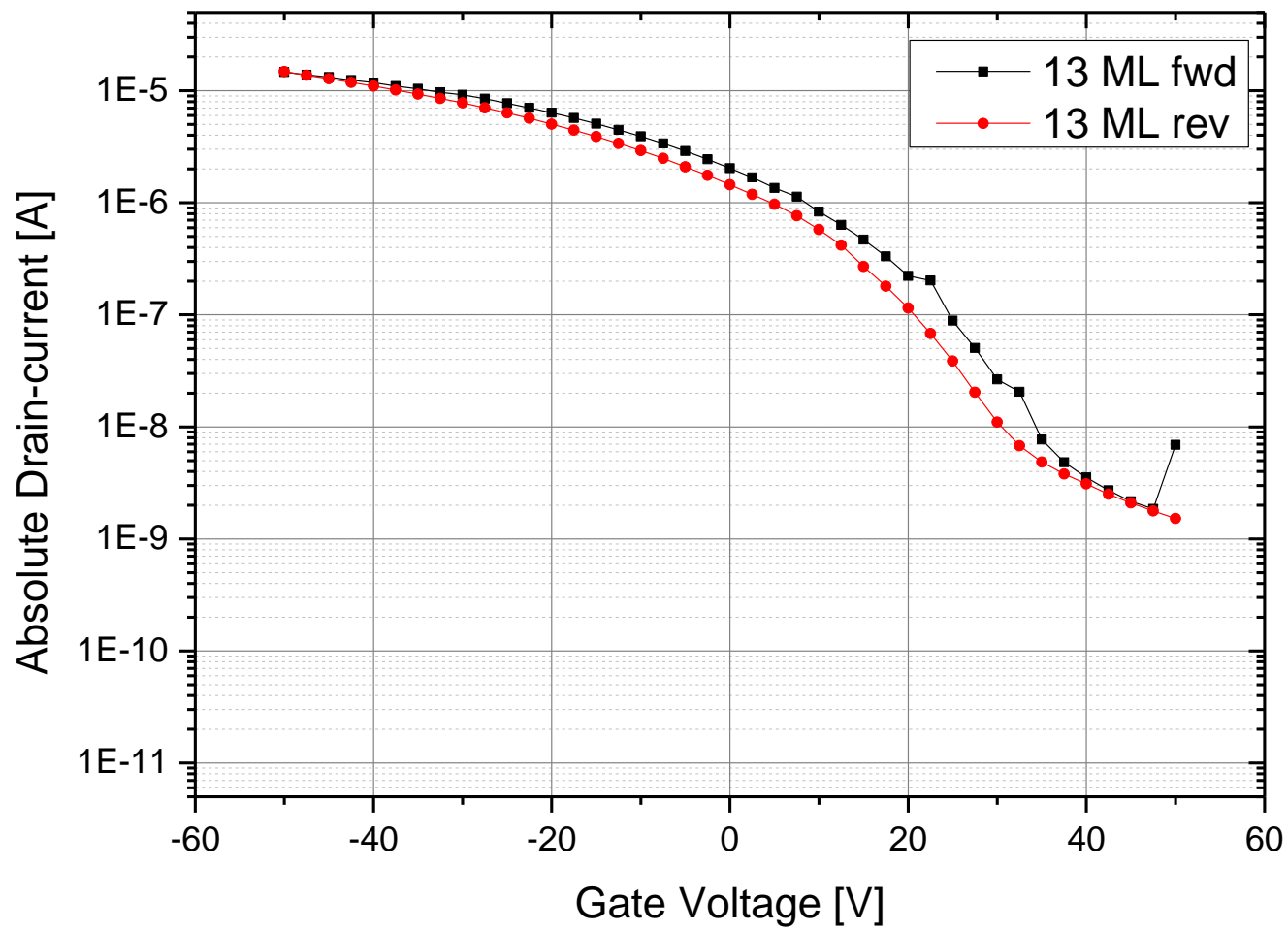

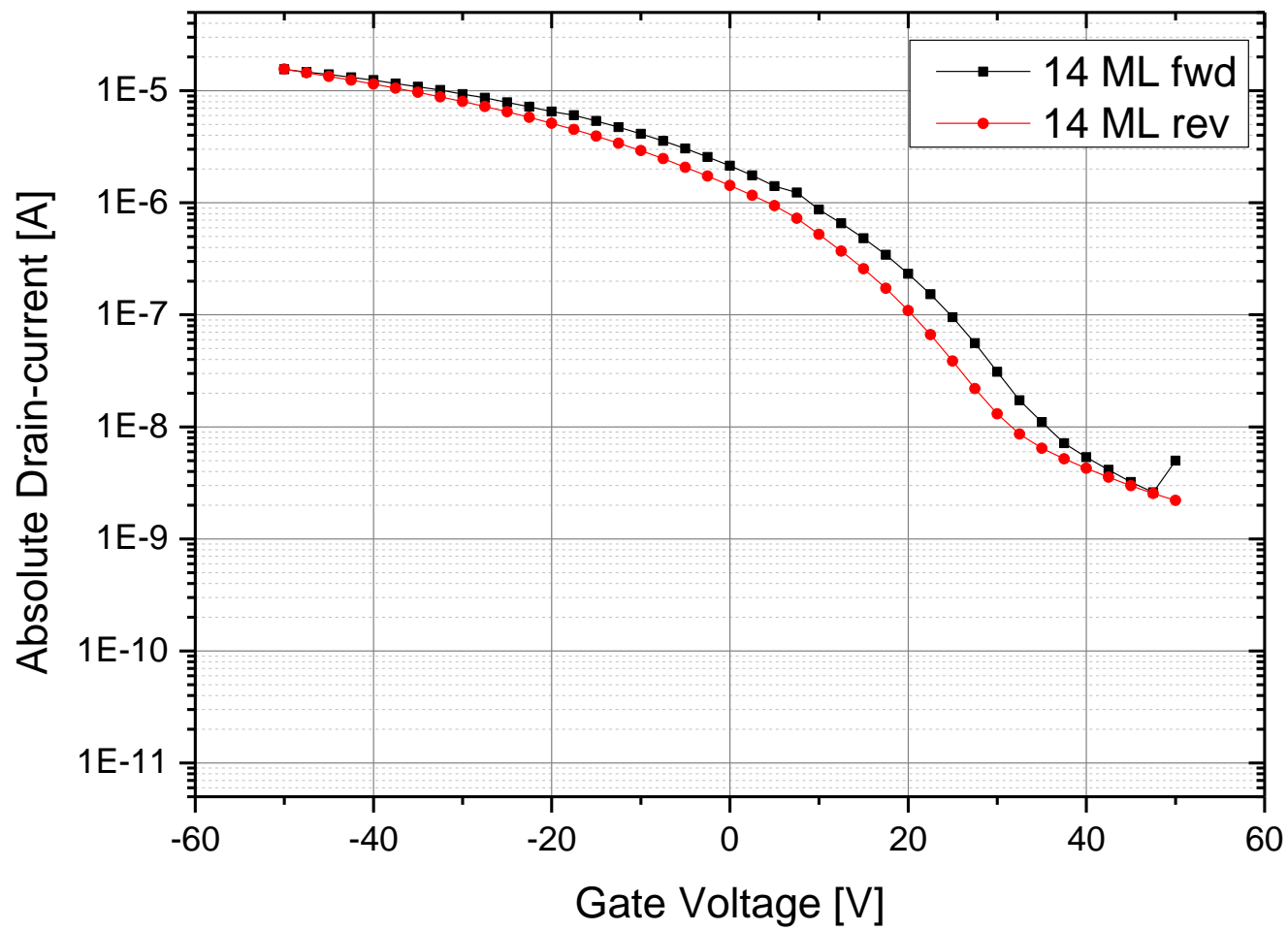

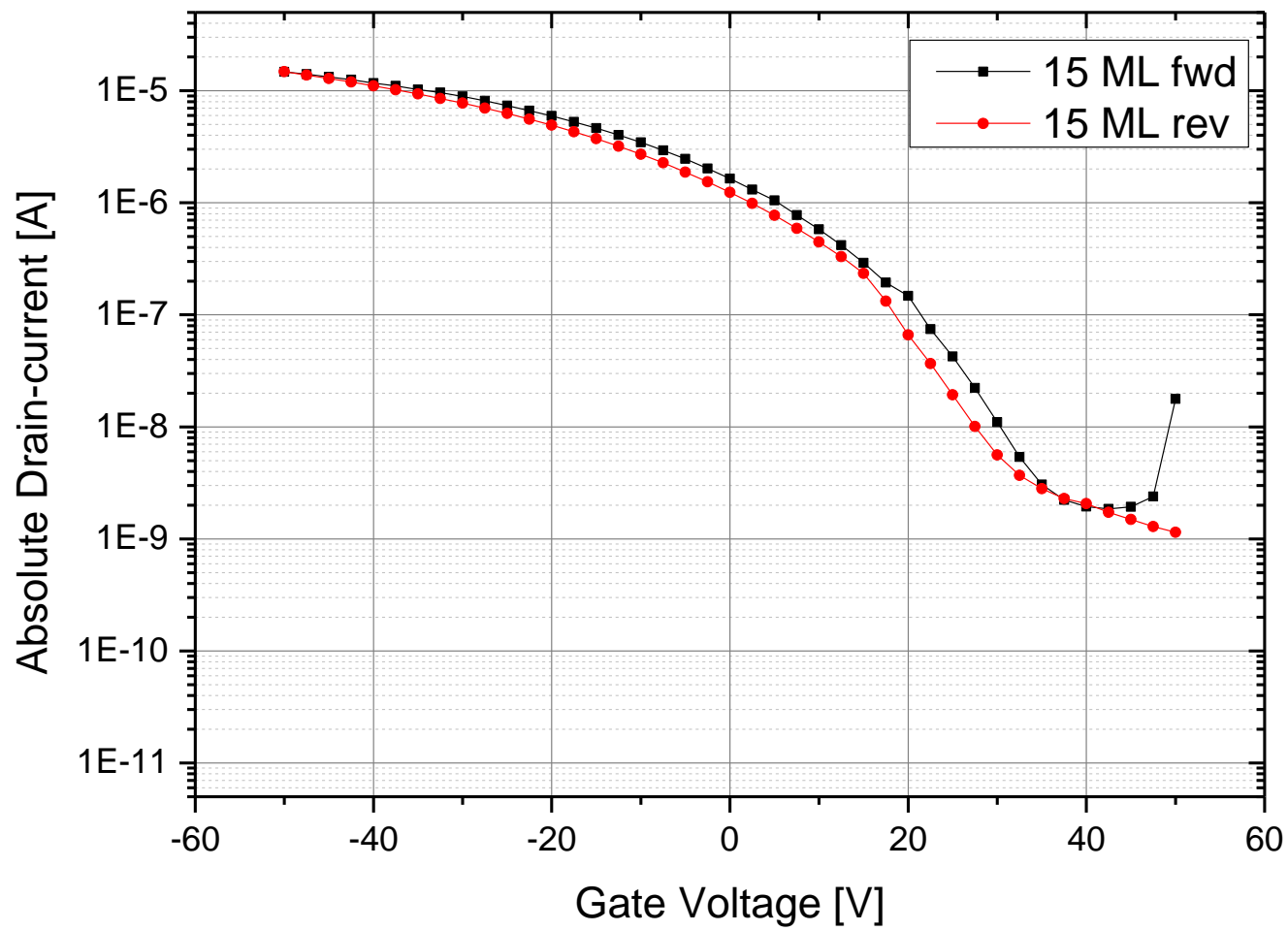

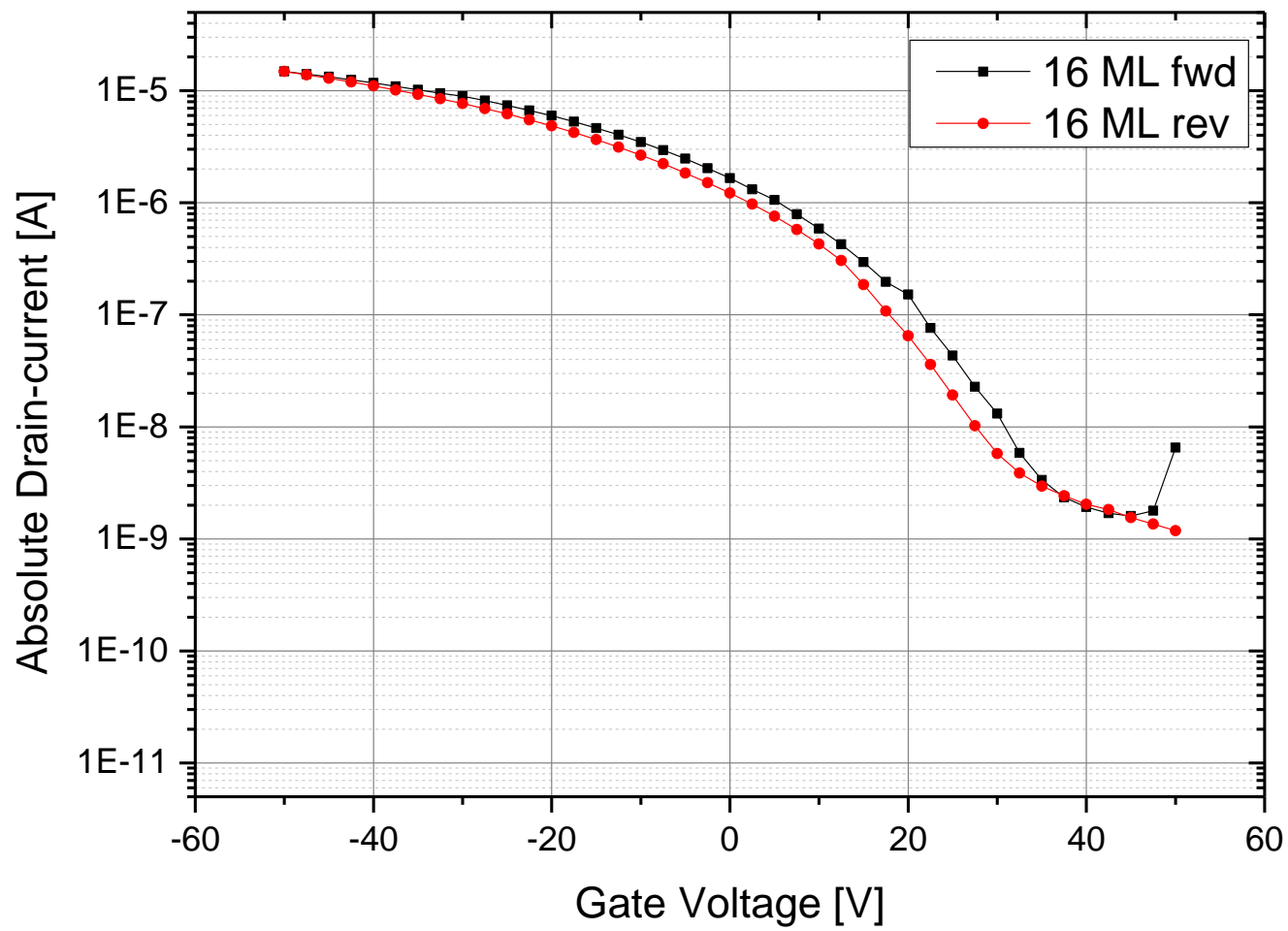

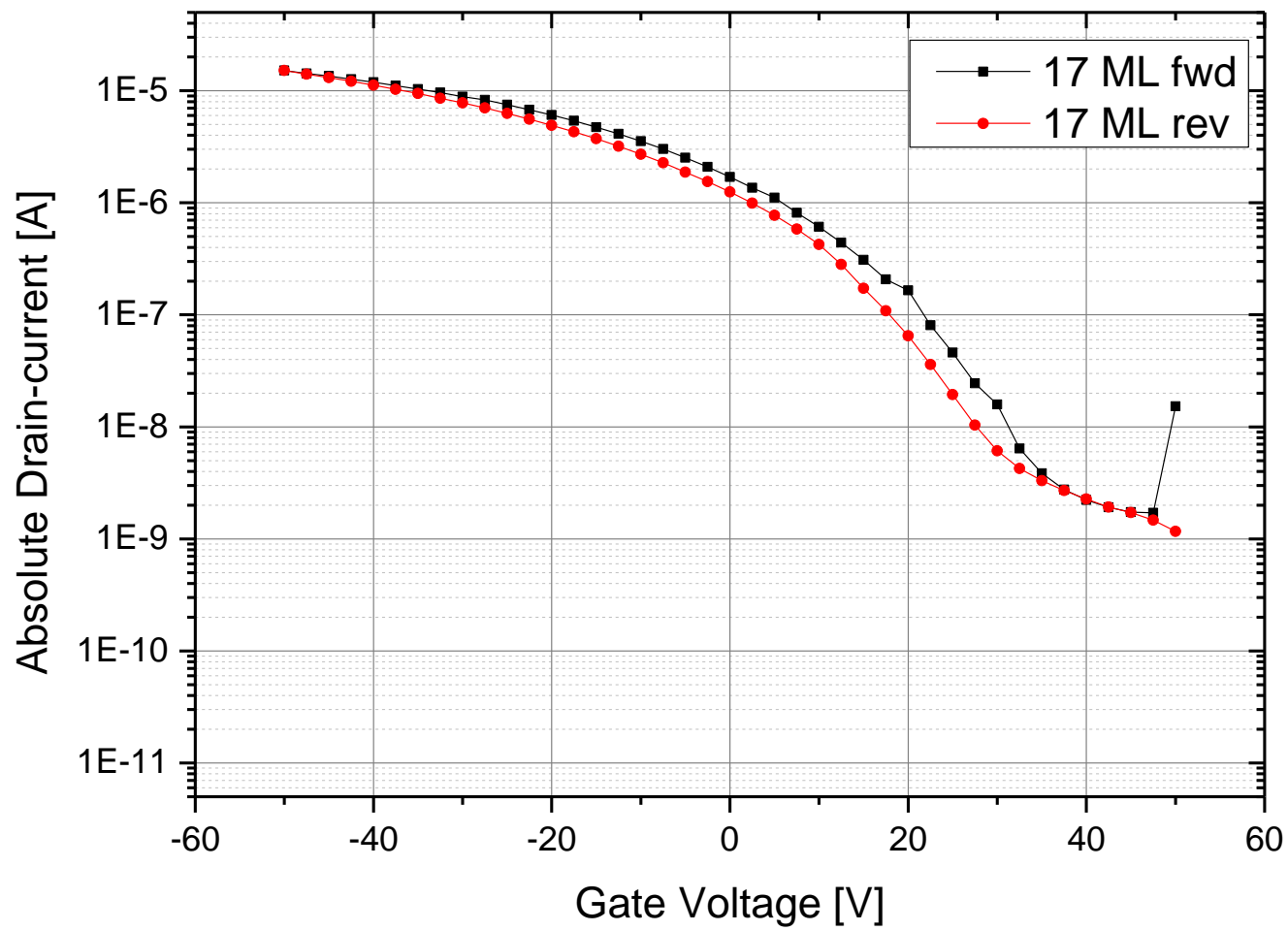

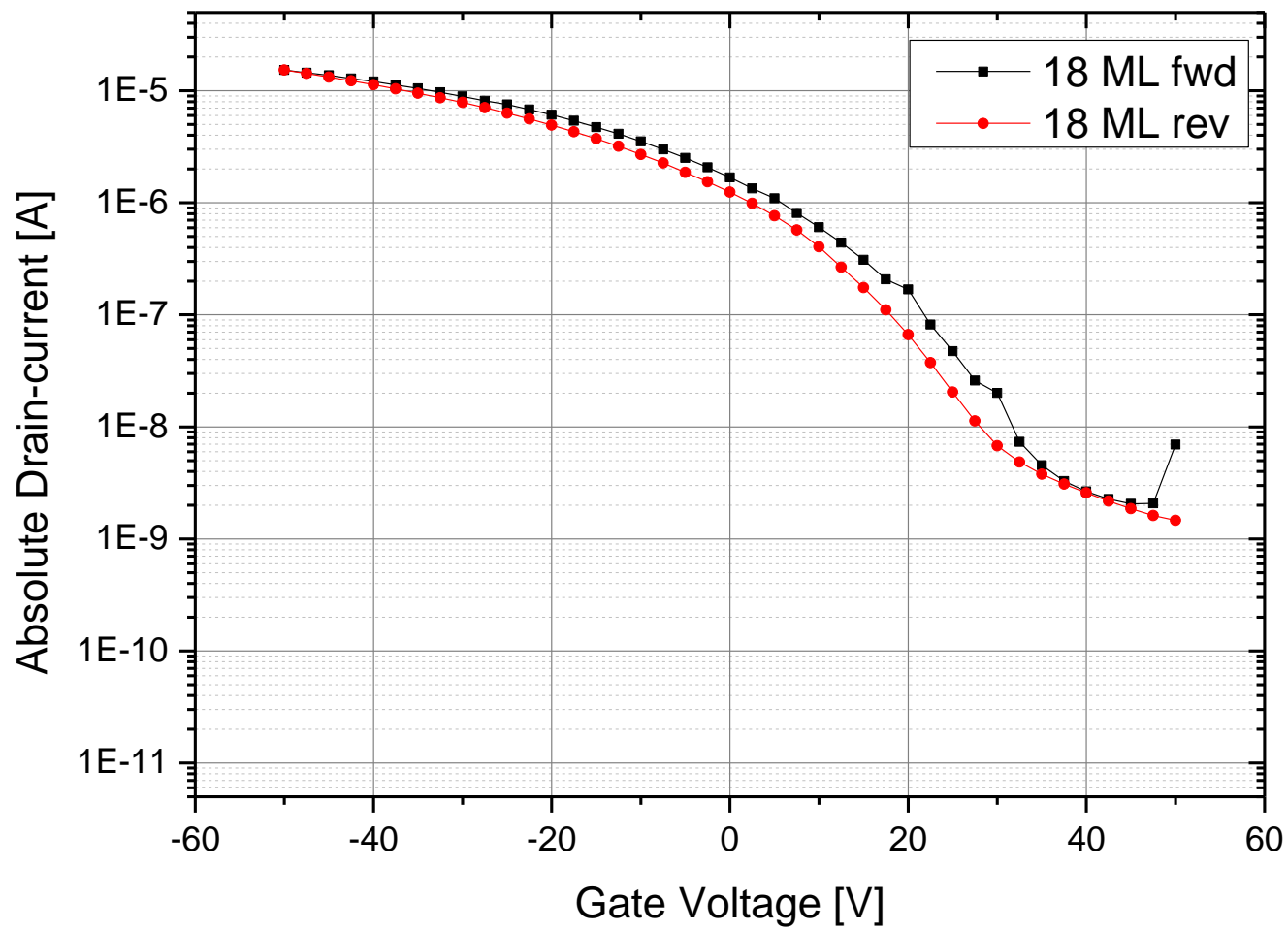

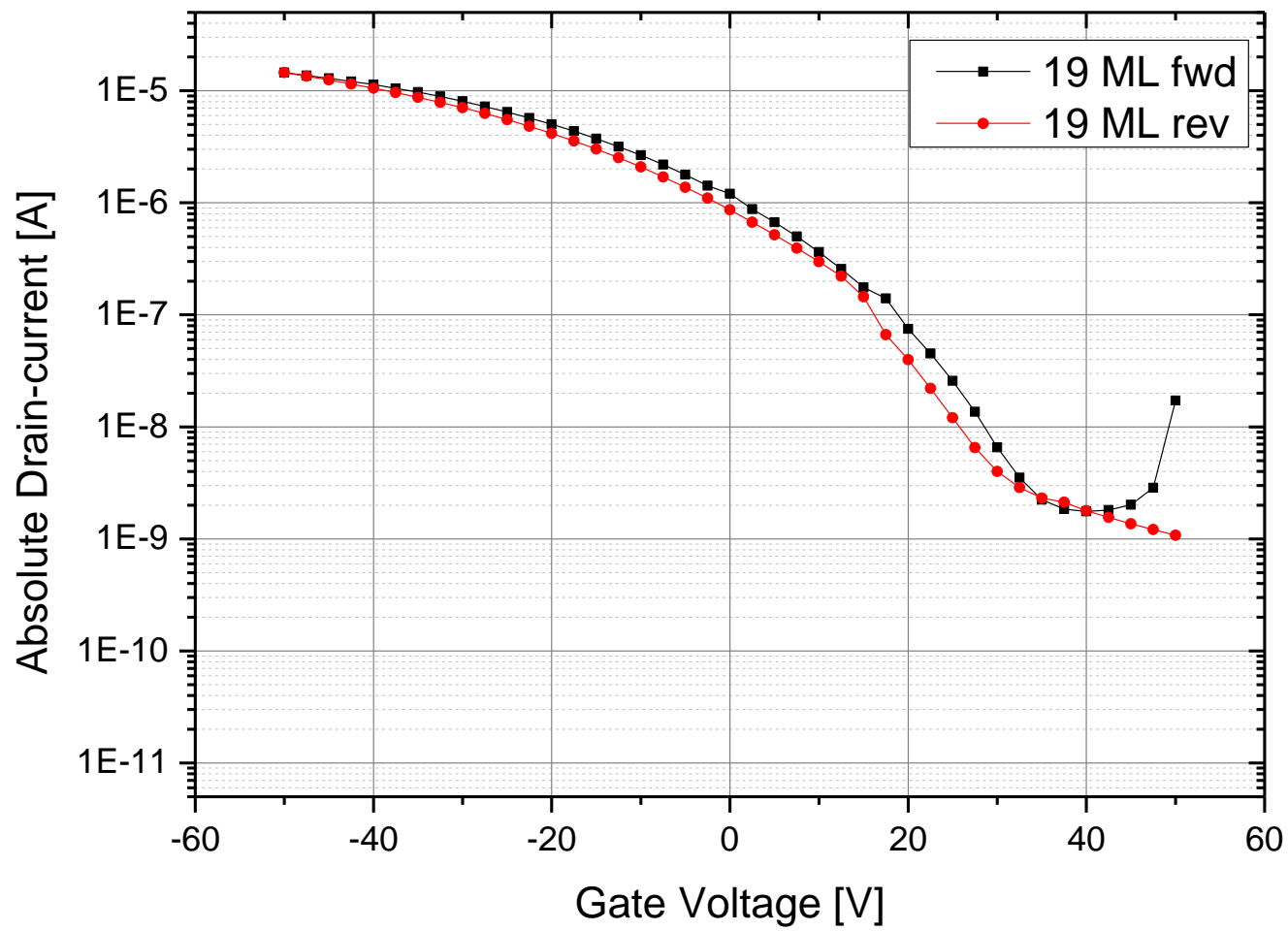

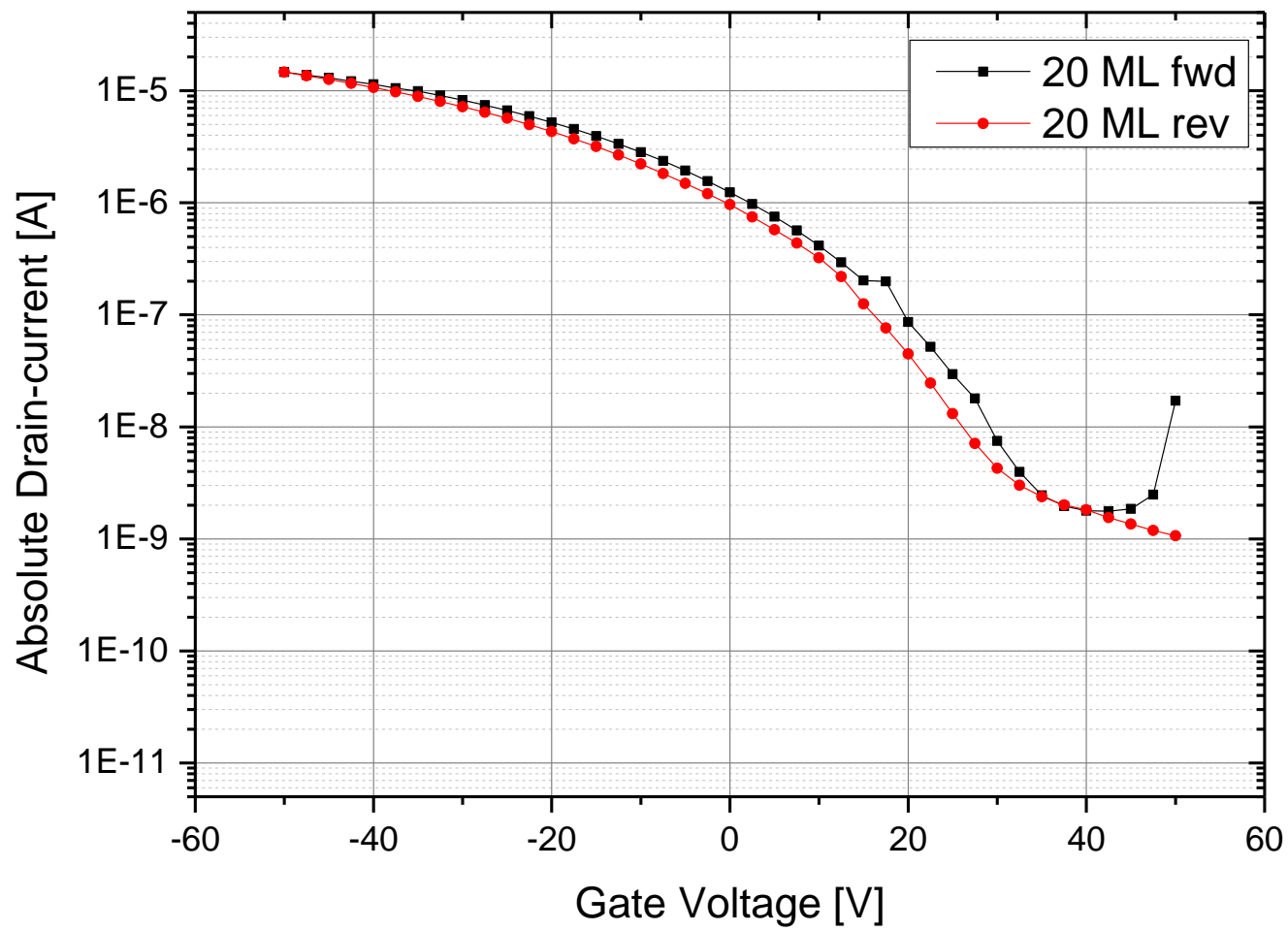

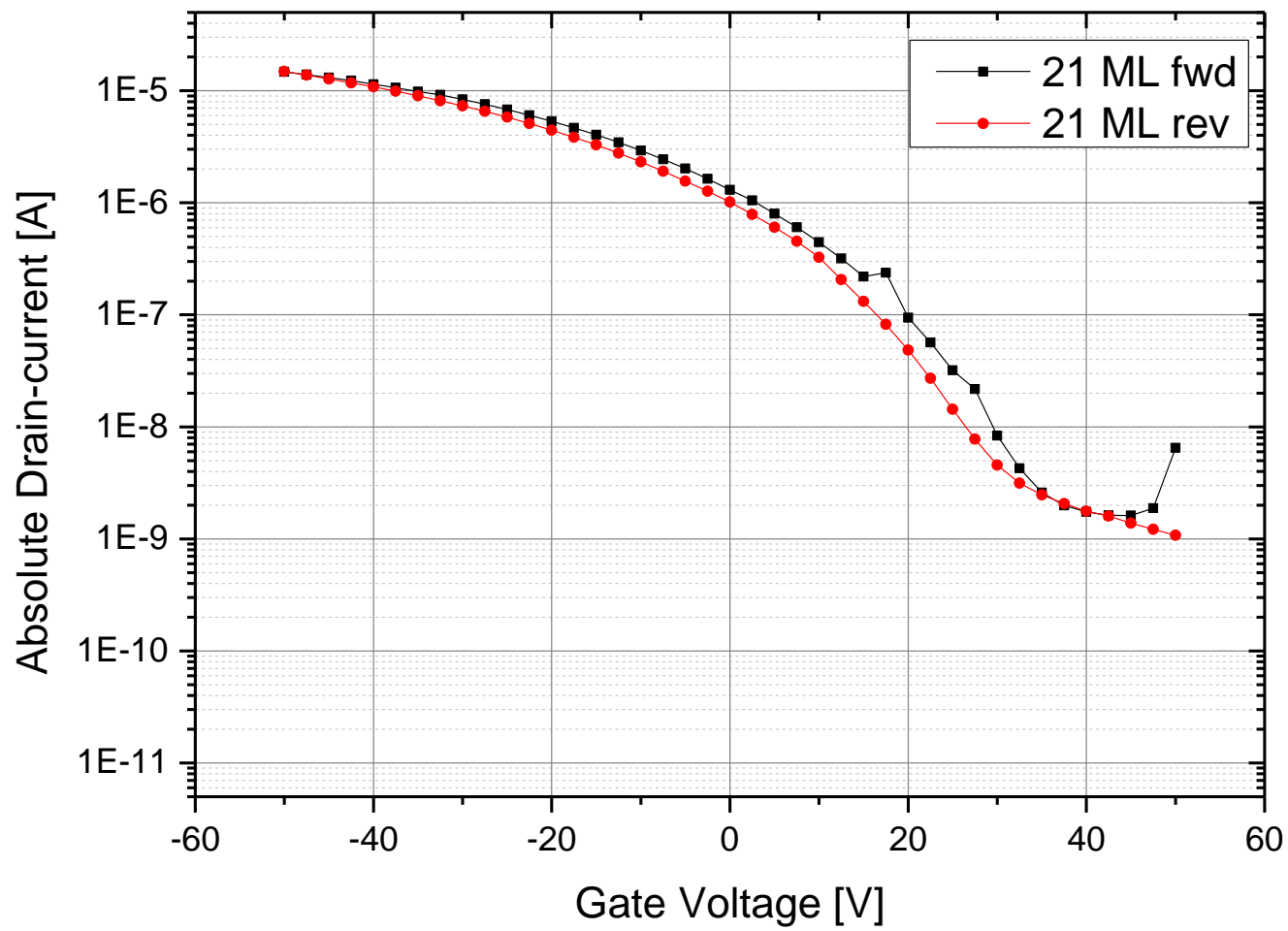

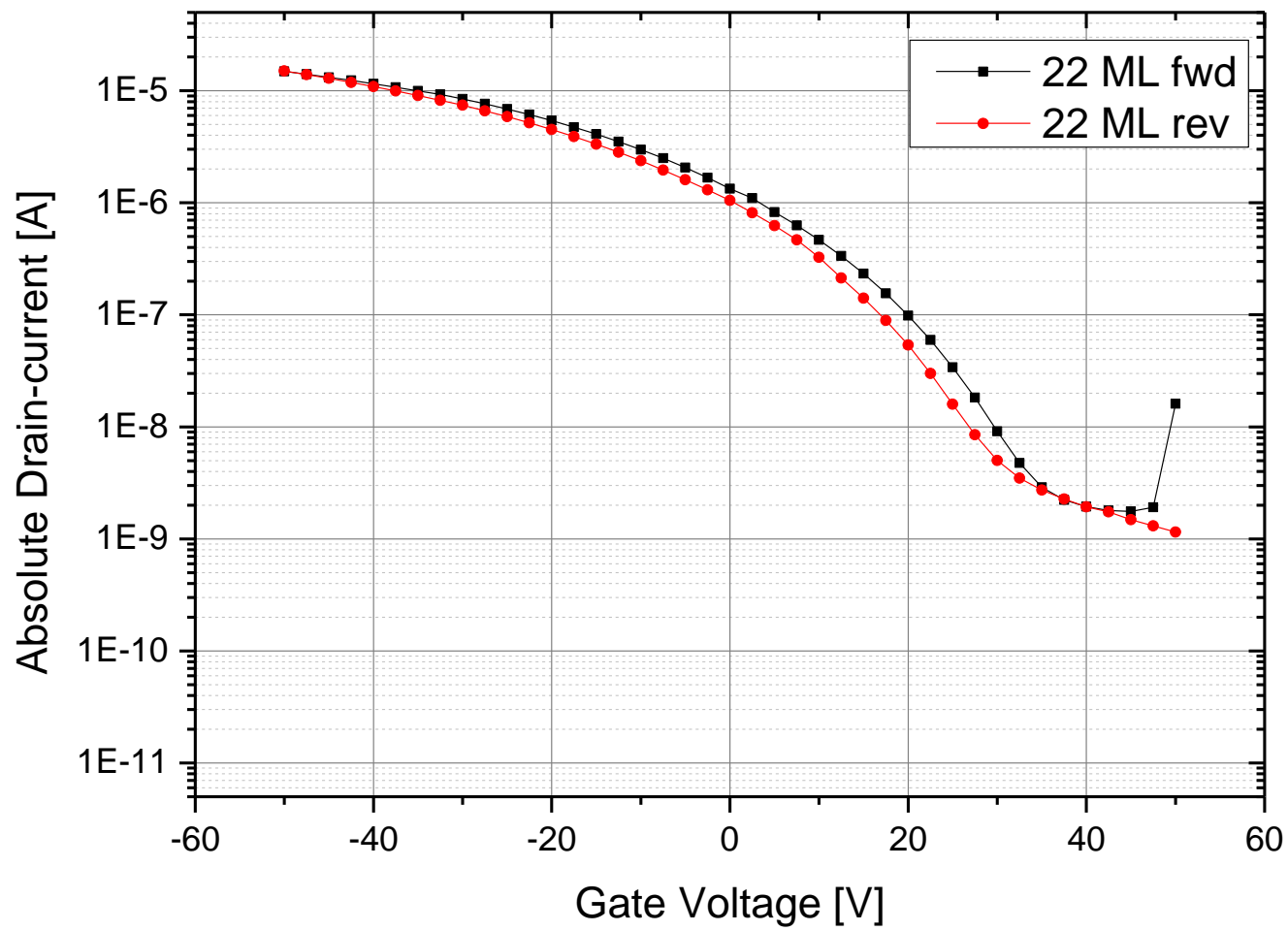

Supplement: Supplementary Information 1 [file NIHMS65285-supplement-Supplementary_Information_1.pdf]
