# Supplementary material for: Electrical in-situ characterisation of interface stabilised organic thin-film transistors
Source: Phys Status Solidi Rapid Res Lett. Author manuscript; Available in PMC 2015 Oct 9. (PMC4599138; doi:10.1002/pssr.201510169)
Supplement: Supplementary Information 2 [file NIHMS65285-supplement-Supplementary_Information_2.pdf]

Pentacene oTFT with SiO_2/Polynorbornene bilayer dielectric

Layer by Layer Transfer Characteristics, Interface Stabilization

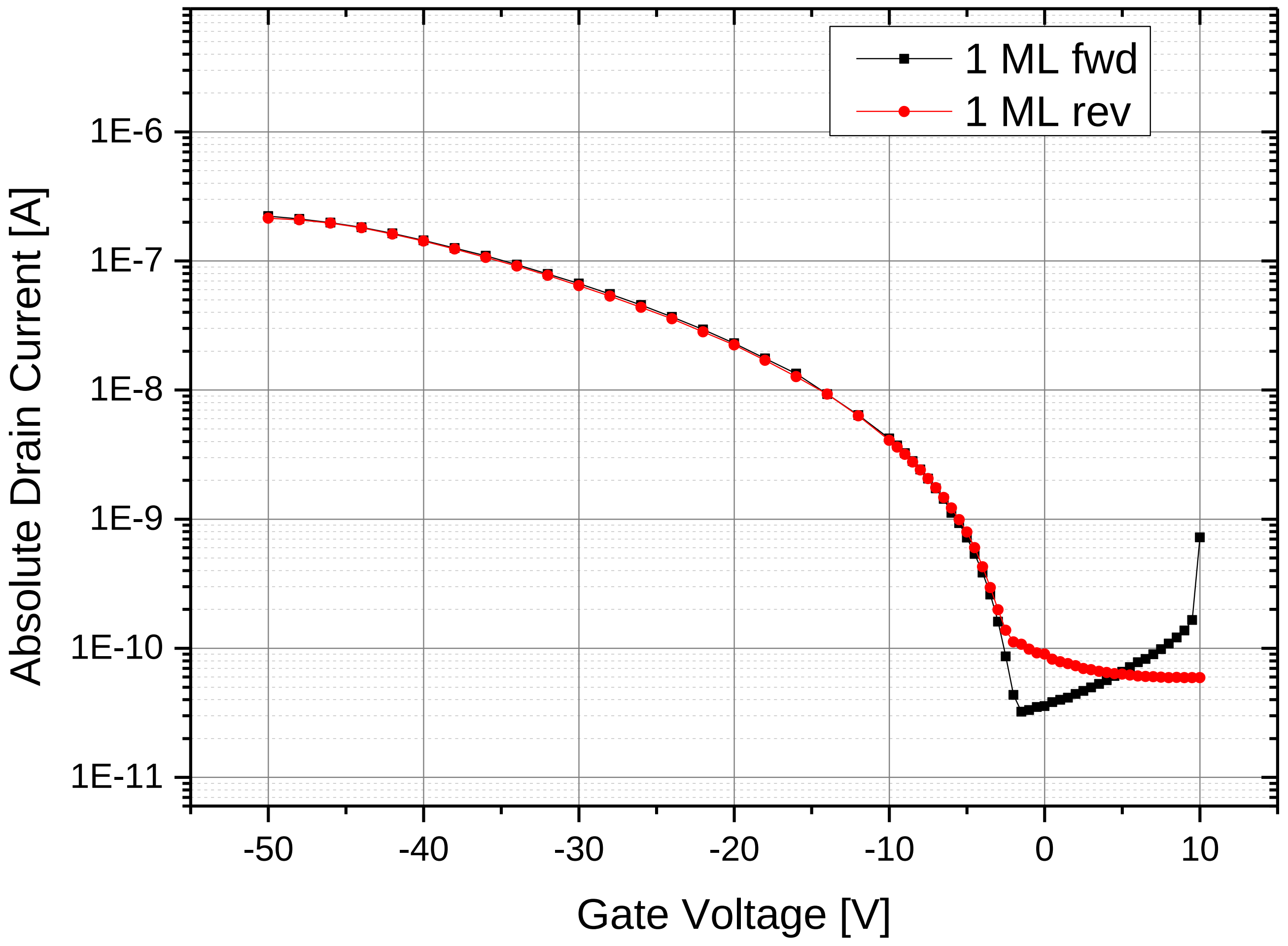

1 ML fwd
1 ML rev
1E-6
1E-7
1E-8
1E-9
1E-10
1E-11
Absolute Drain Current [A]
-50
-40
-30
-20
-10
0
10
Gate Voltage [V]

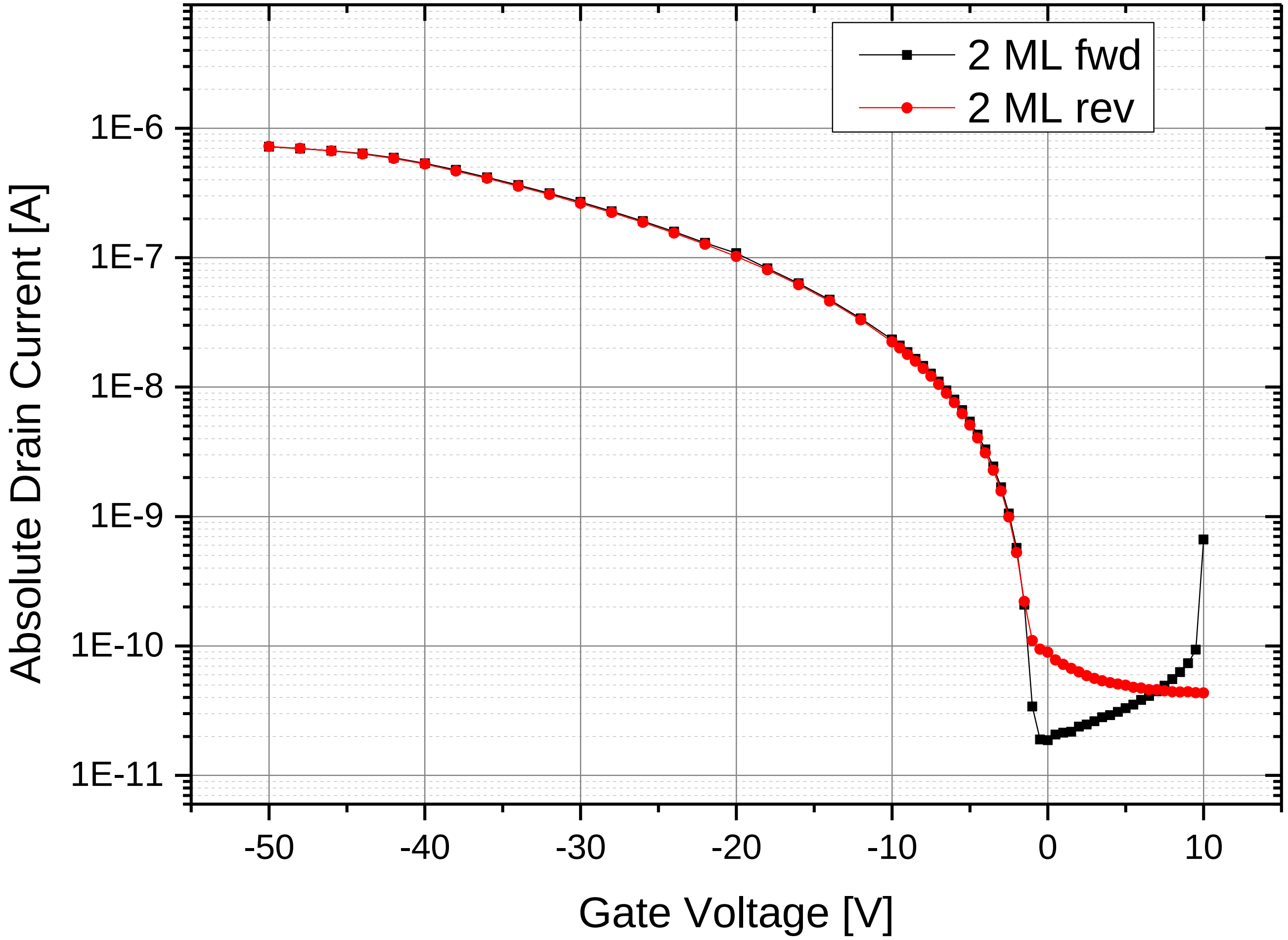

2 ML fwd
2 ML rev
Absolute Drain Current [A]
1E-6
1E-7
1E-8
1E-9
1E-10
1E-11
-50
-40
-30
-20
-10
0
10
Gate Voltage [V]

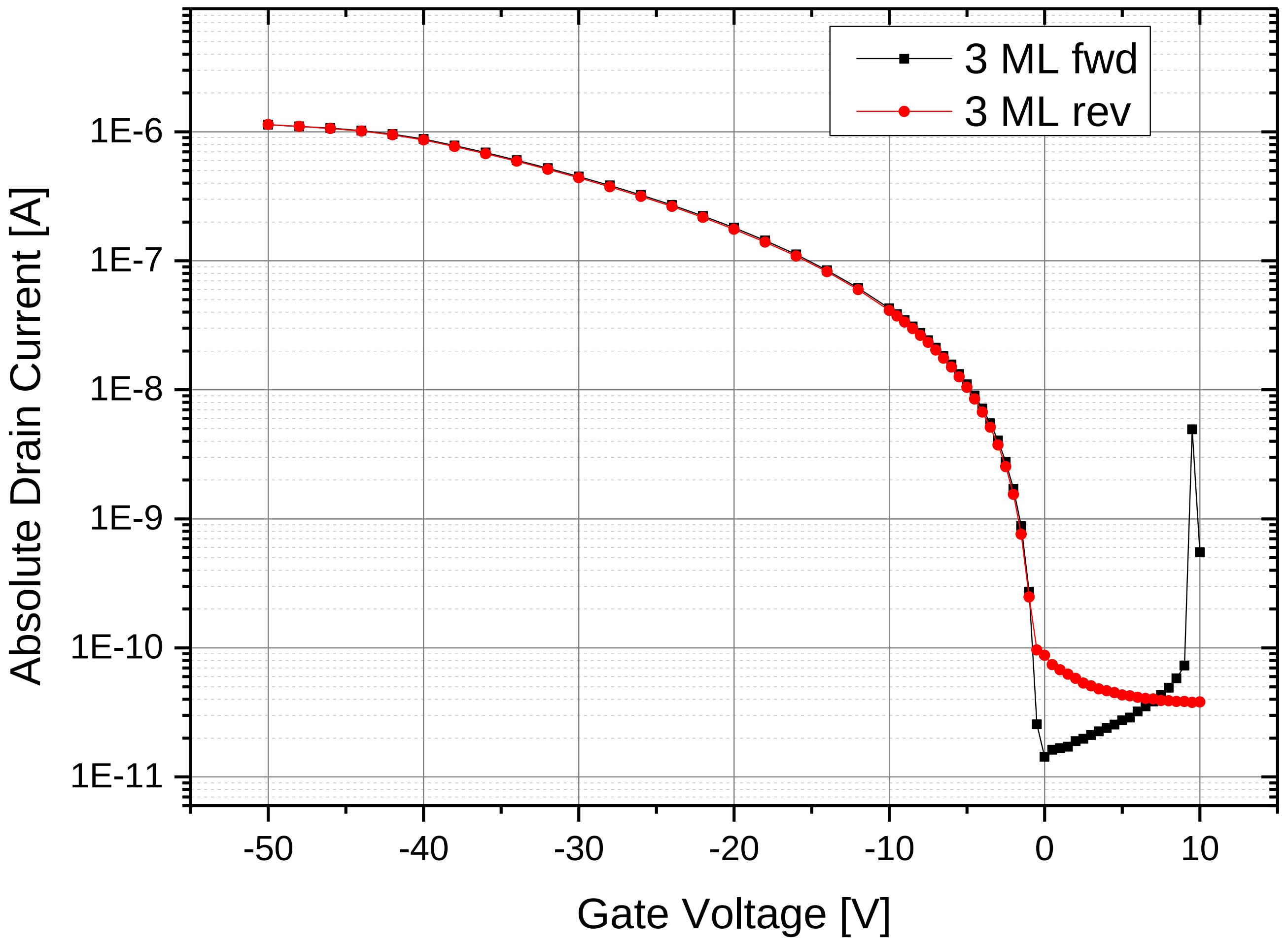

3 ML fwd
3 ML rev
1E-6
1E-7
1E-8
1E-9
1E-10
1E-11
Absolute Drain Current [A]
-50
-40
-30
-20
-10
0
10
Gate Voltage [V]

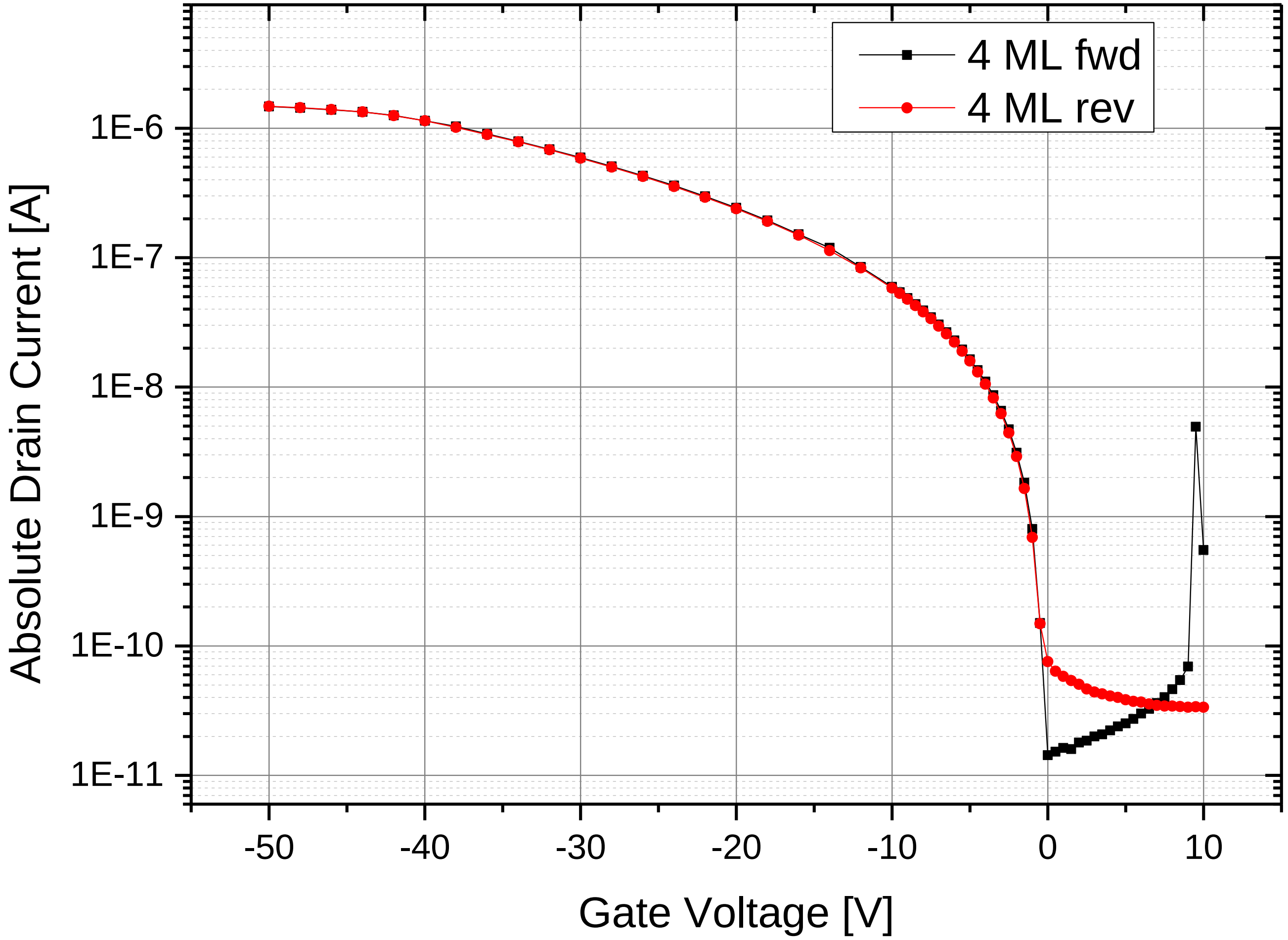

4 ML fwd
4 ML rev
Absolute Drain Current [A]
1E-6
1E-7
1E-8
1E-9
1E-10
1E-11
-50
-40
-30
-20
-10
0
10
Gate Voltage [V]

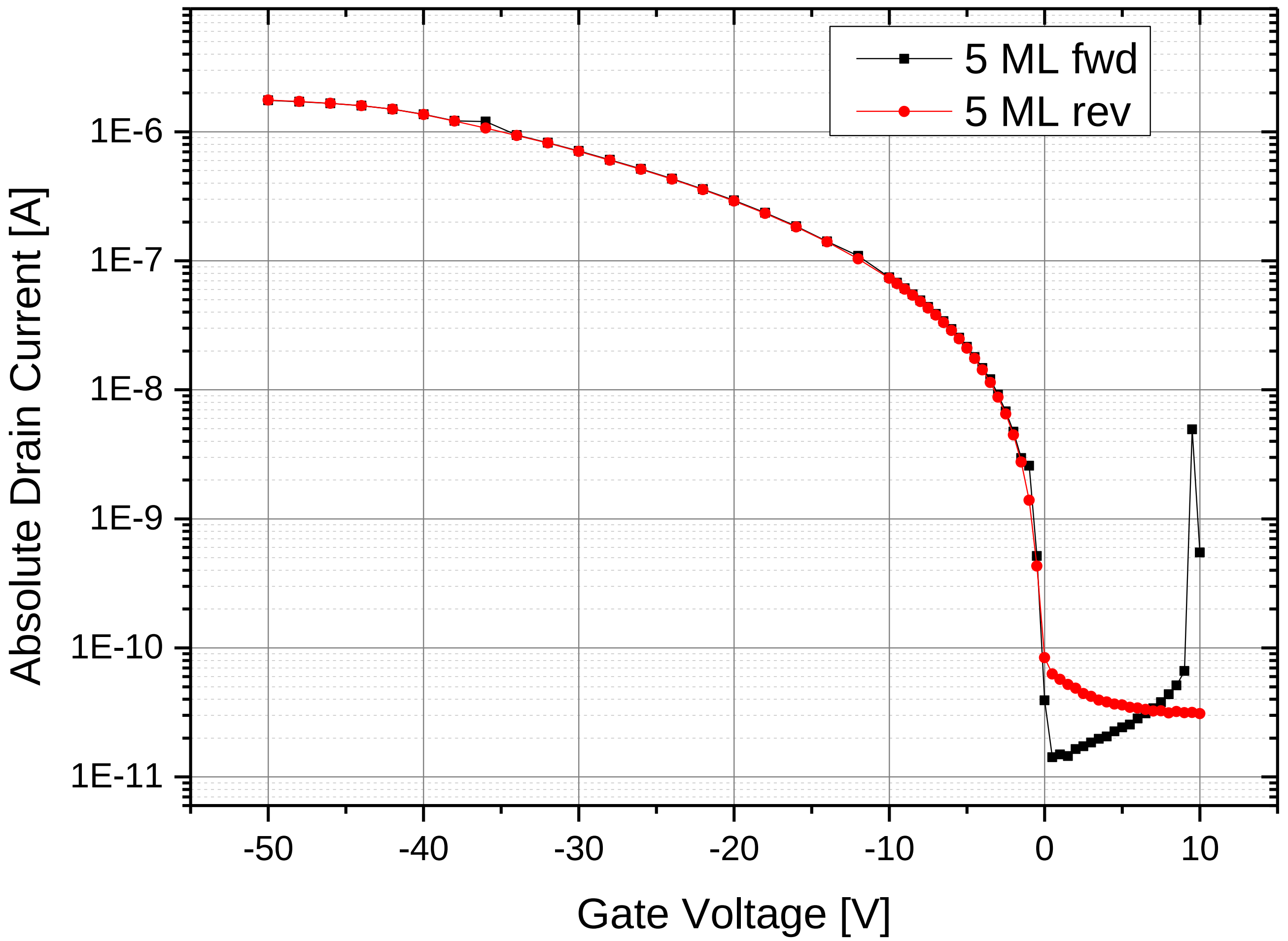

5 ML fwd
5 ML rev
Absolute Drain Current [A]
1E-6
1E-7
1E-8
1E-9
1E-10
1E-11
-50
-40
-30
-20
-10
0
10
Gate Voltage [V]

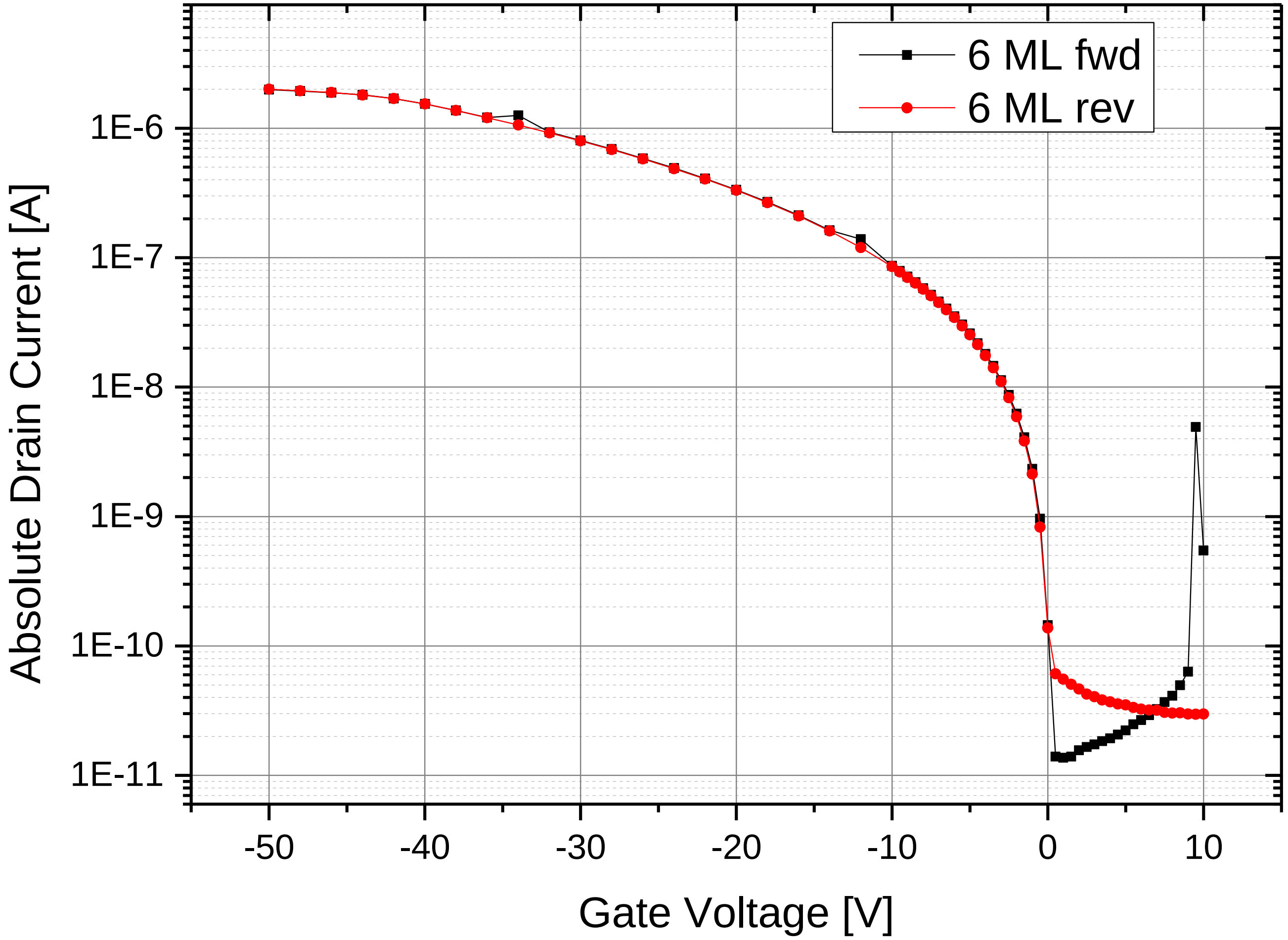

6 ML fwd
6 ML rev
Absolute Drain Current [A]
1E-6
1E-7
1E-8
1E-9
1E-10
1E-11
-50
-40
-30
-20
-10
0
10
Gate Voltage [V]

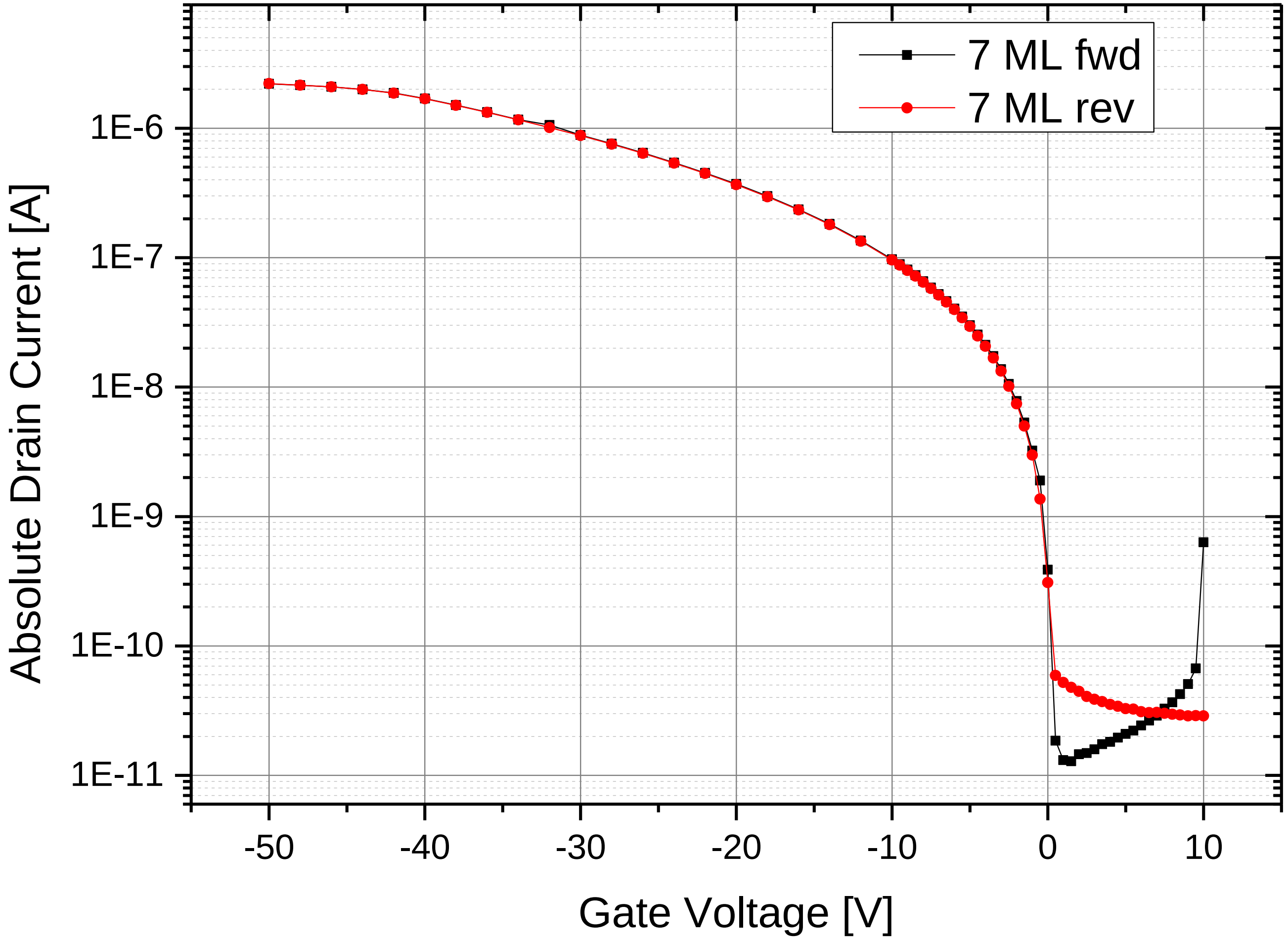

7 ML fwd
7 ML rev
Absolute Drain Current [A]
1E-6
1E-7
1E-8
1E-9
1E-10
1E-11
-50
-40
-30
-20
-10
0
10
Gate Voltage [V]

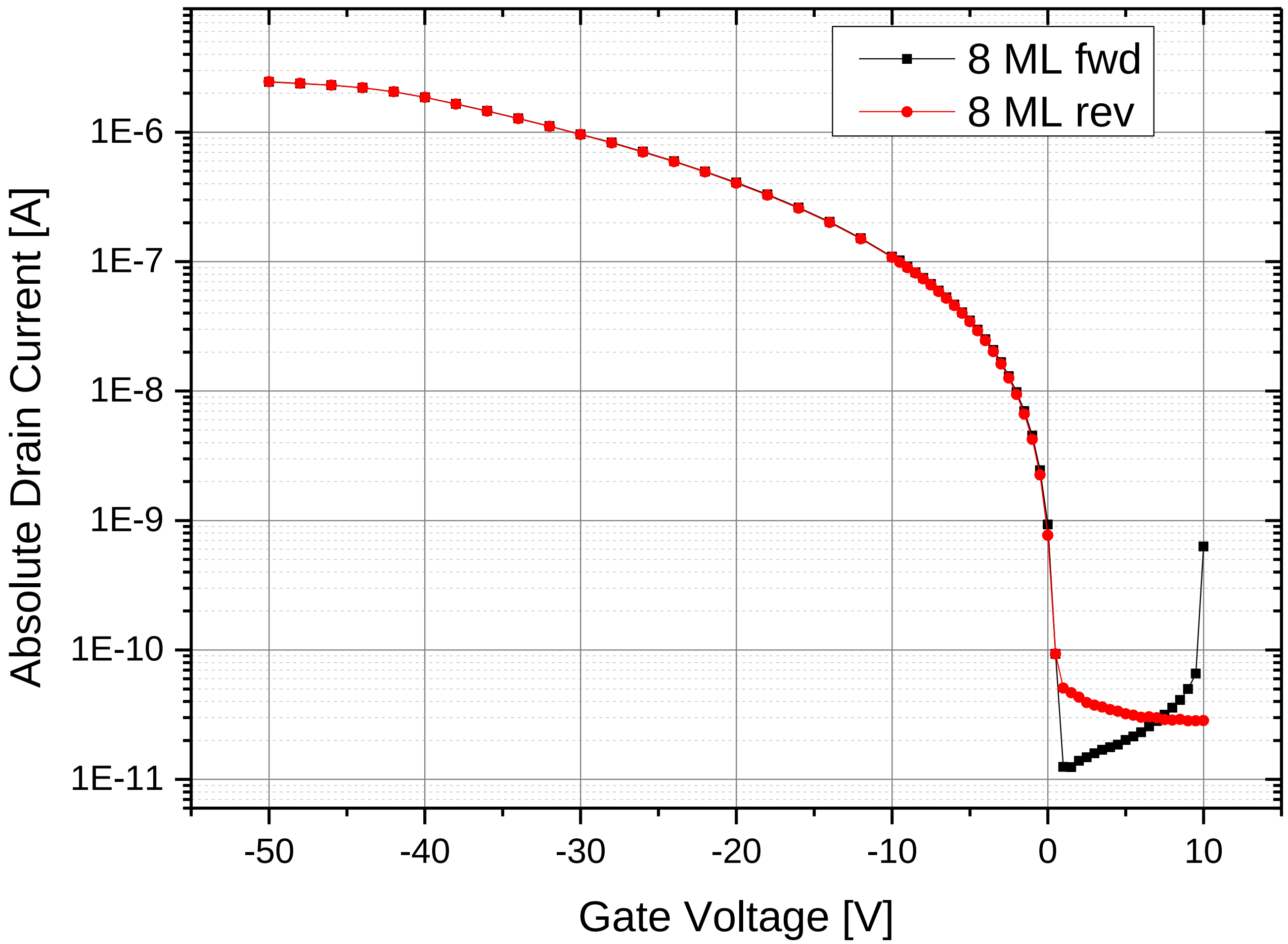
8 ML fwd
8 ML rev
Absolute Drain Current [A]
1E-6
1E-7
1E-8
1E-9
1E-10
1E-11
-50
-40
-30
-20
-10
0
10
Gate Voltage [V]

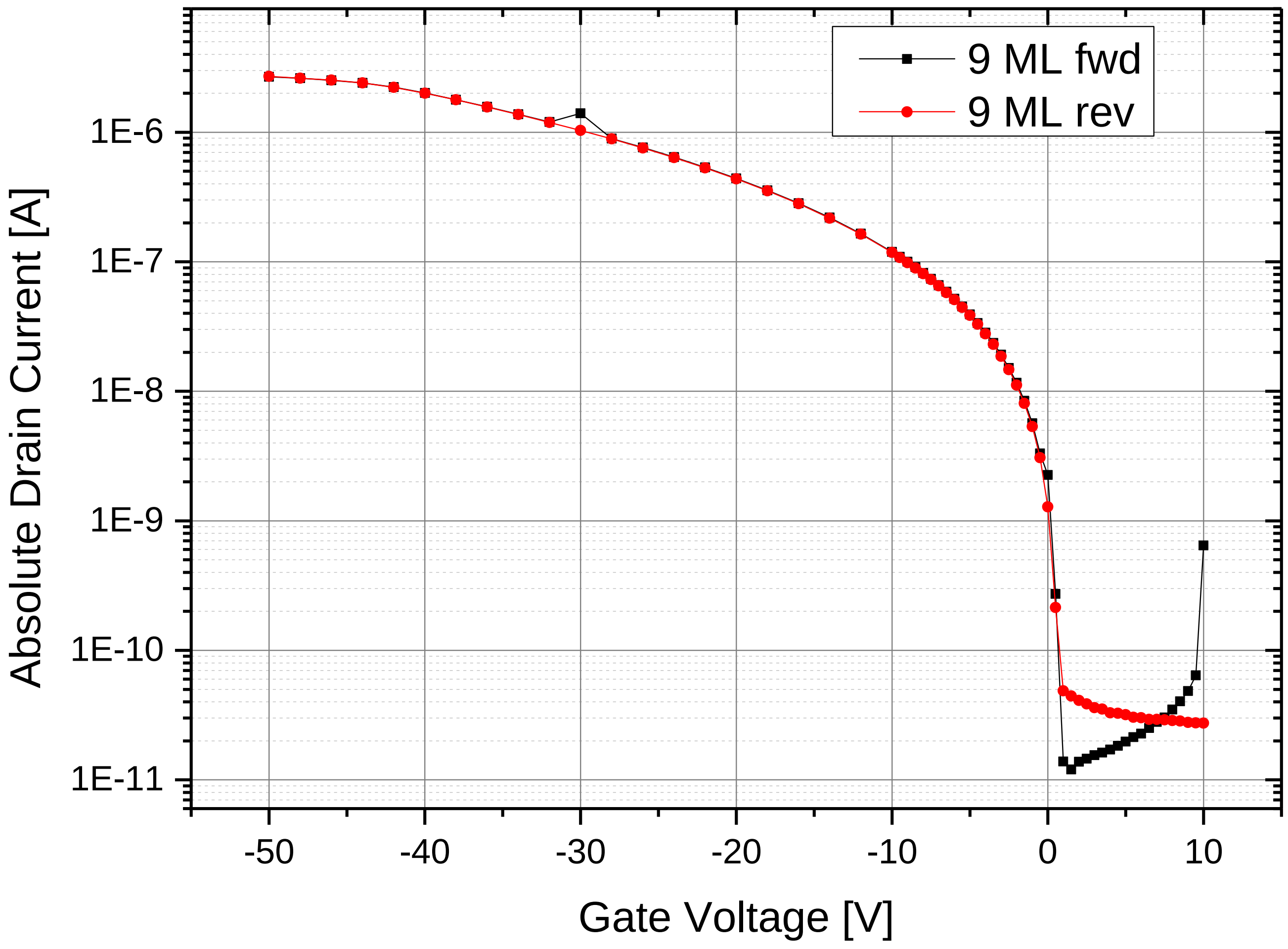
9 ML fwd
9 ML rev
1E-6
1E-7
1E-8
1E-9
1E-10
1E-11
-50
-40
-30
-20
-10
0
10
Absolute Drain Current [A]
Gate Voltage [V]

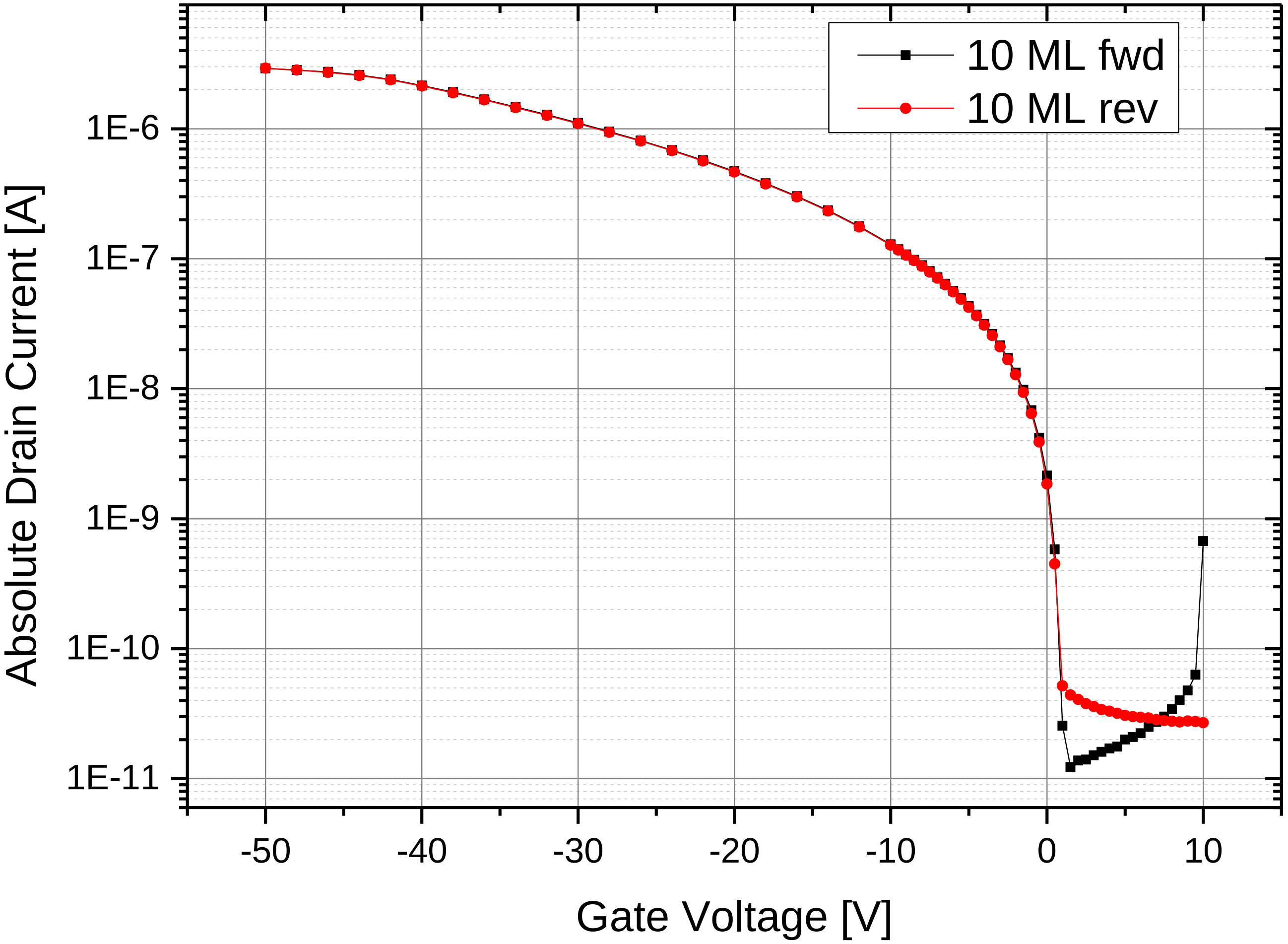

10 ML fwd
10 ML rev
1E-6
1E-7
1E-8
1E-9
1E-10
1E-11
Absolute Drain Current [A]
-50
-40
-30
-20
-10
0
10
Gate Voltage [V]

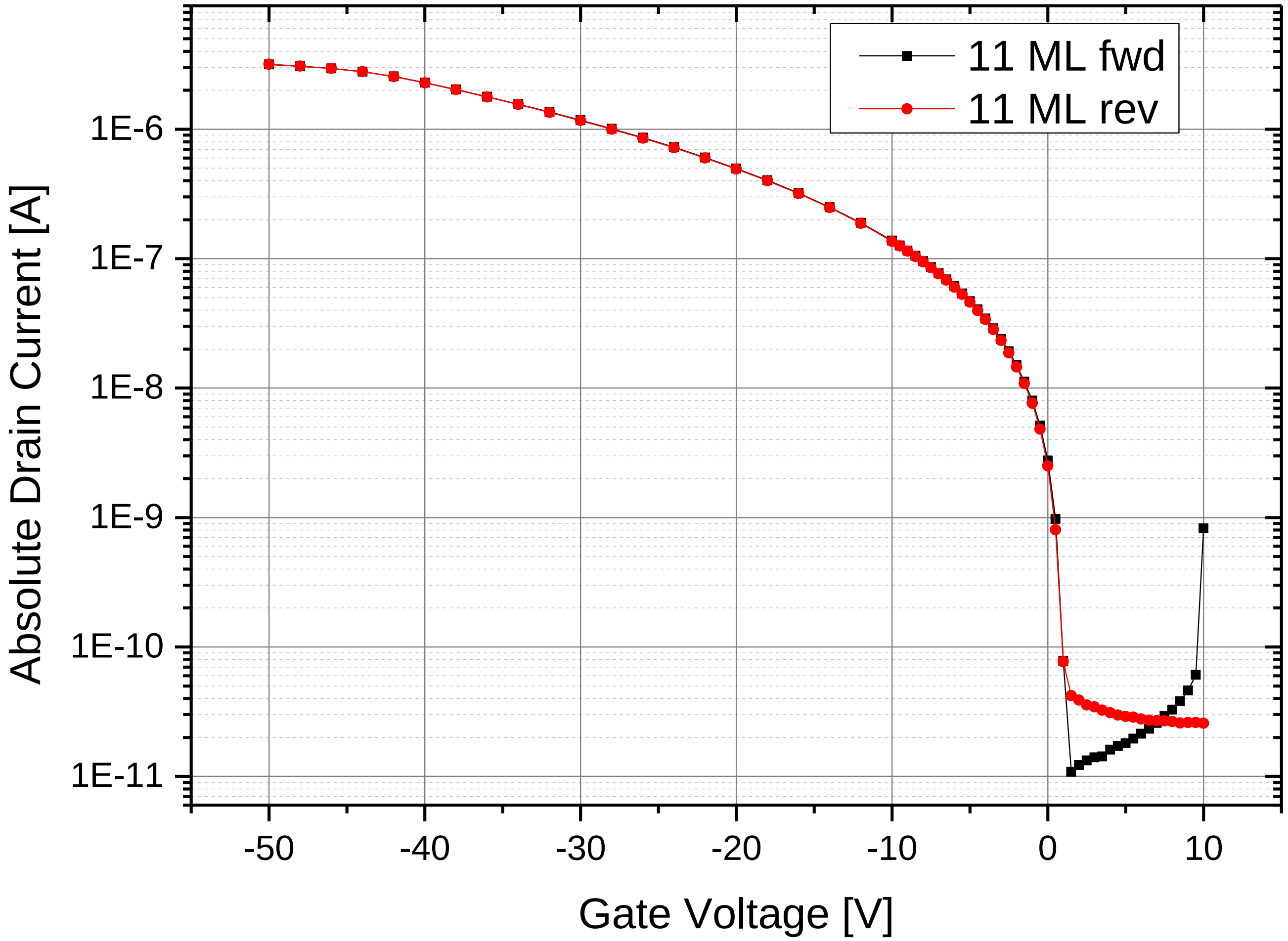
11 ML fwd
11 ML rev
1E-6
1E-7
1E-8
1E-9
1E-10
1E-11
Absolute Drain Current [A]
-50
-40
-30
-20
-10
0
10
Gate Voltage [V]

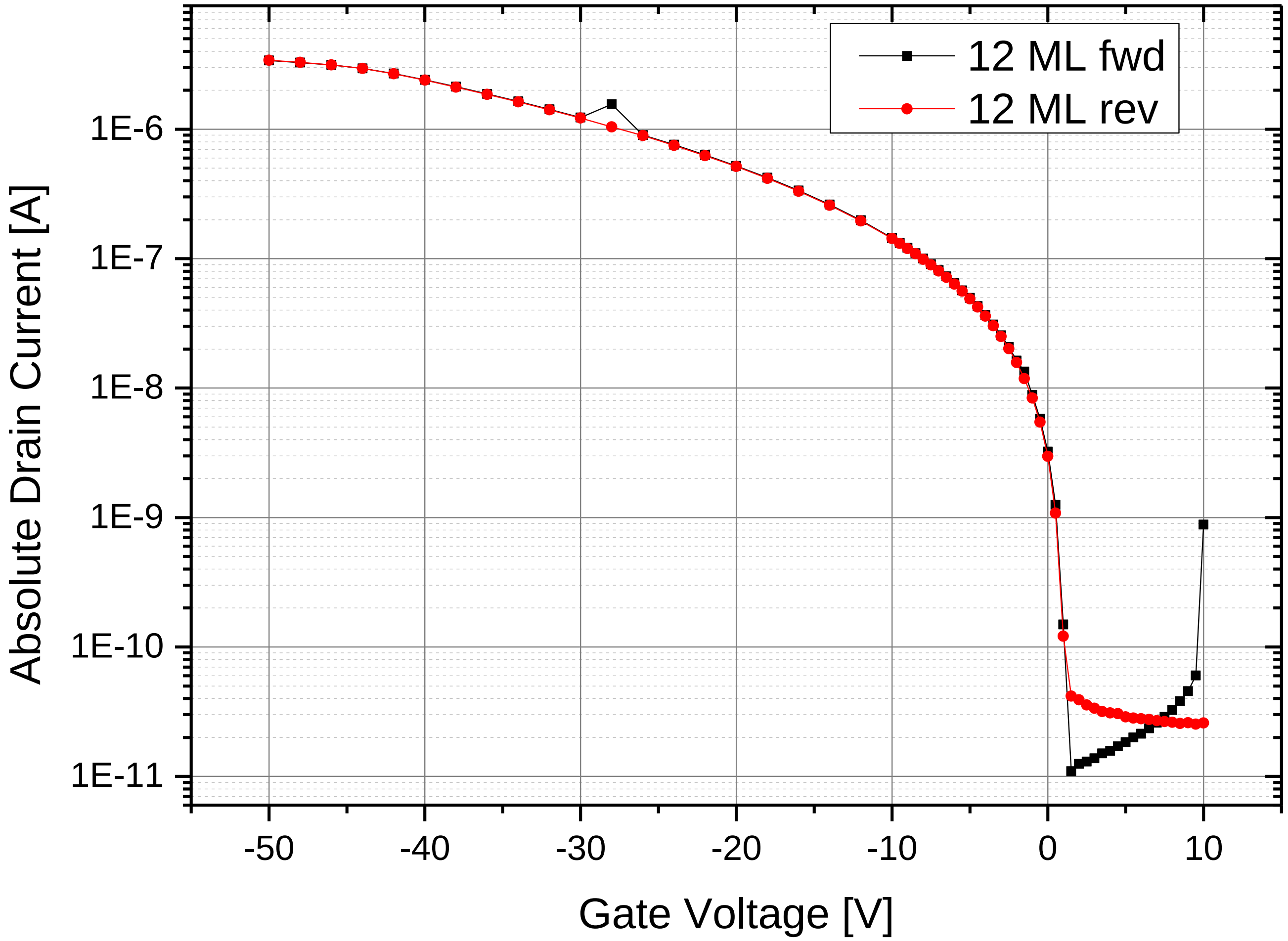

12 ML fwd
12 ML rev
1E-6
1E-7
1E-8
1E-9
1E-10
1E-11
Absolute Drain Current [A]
-50
-40
-30
-20
-10
0
10
Gate Voltage [V]

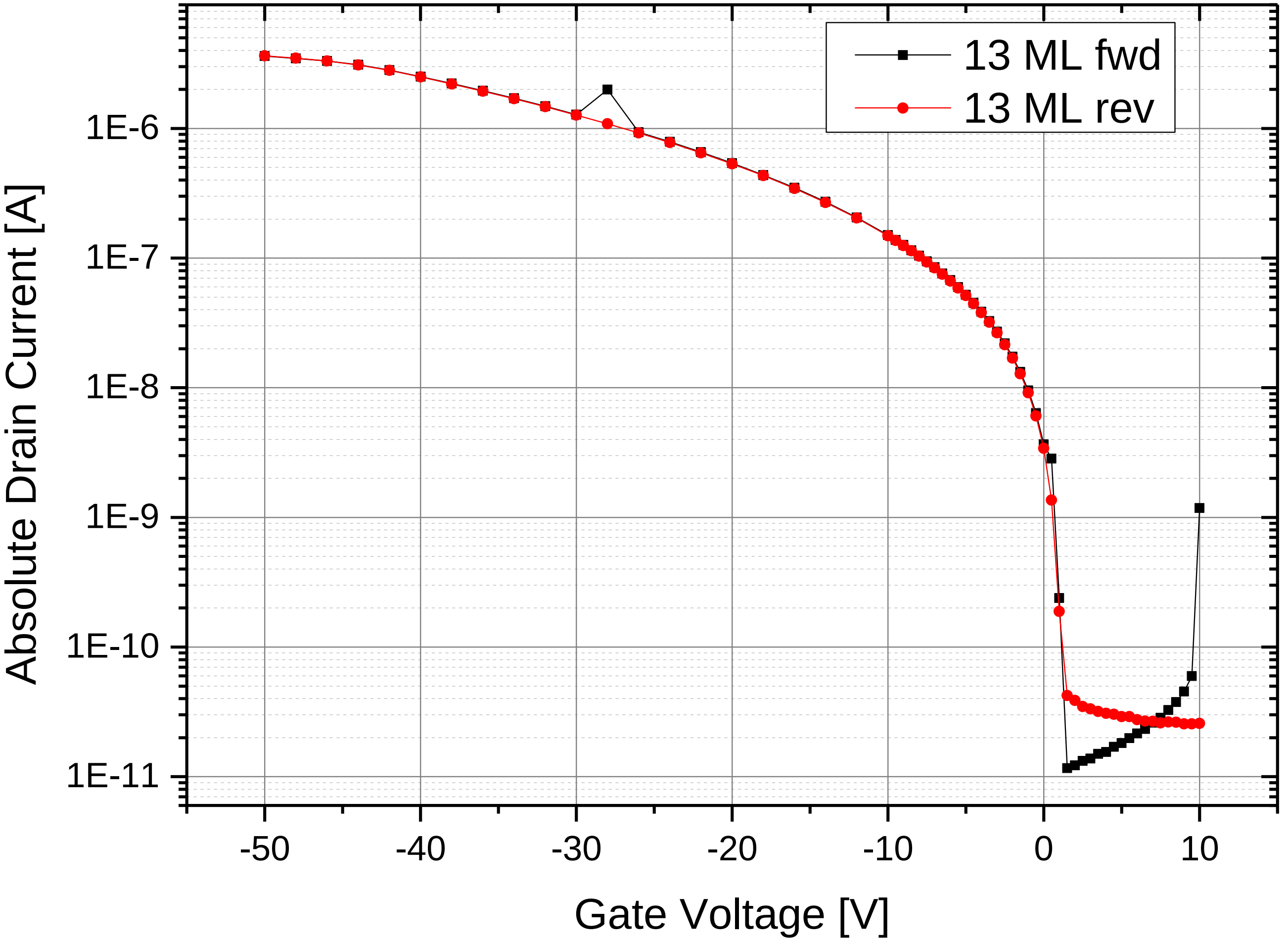
13 ML fwd
13 ML rev
Absolute Drain Current [A]
1E-6
1E-7
1E-8
1E-9
1E-10
1E-11
-50
-40
-30
-20
-10
0
10
Gate Voltage [V]

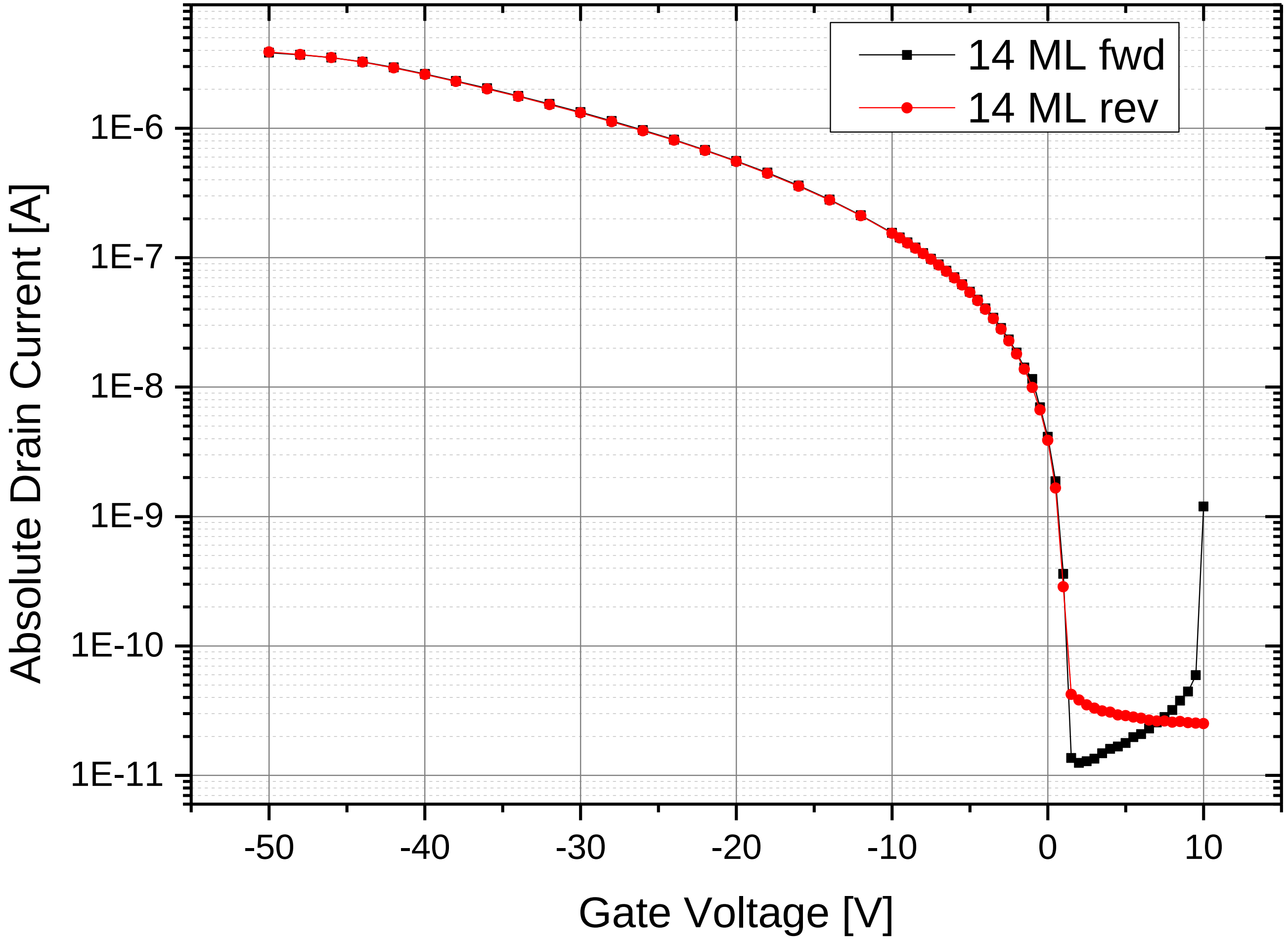

14 ML fwd
14 ML rev
1E-6
1E-7
1E-8
1E-9
1E-10
1E-11
Absolute Drain Current [A]
-50
-40
-30
-20
-10
0
10
Gate Voltage [V]

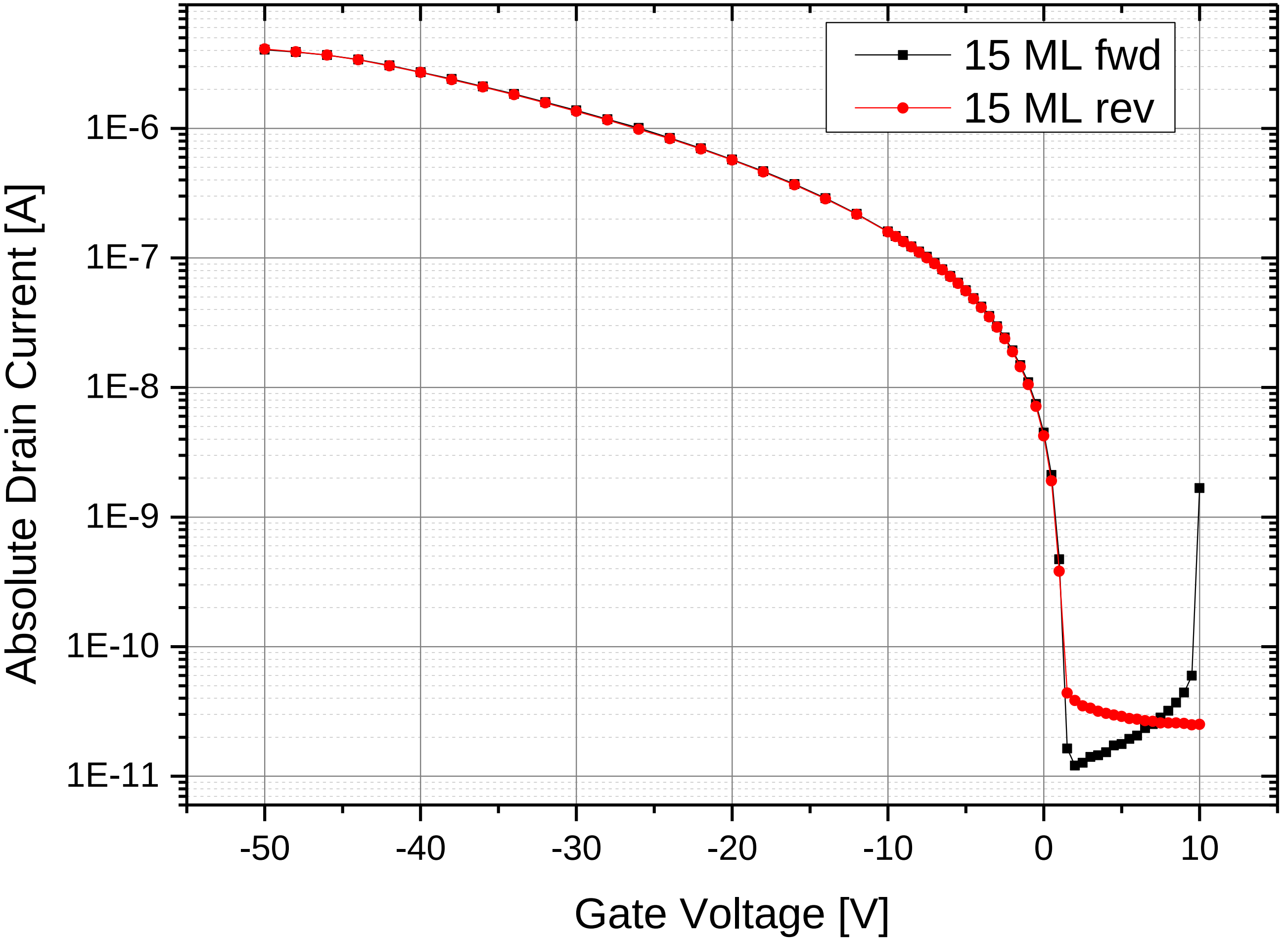
15 ML fwd
15 ML rev
1E-6
1E-7
1E-8
1E-9
1E-10
1E-11
Absolute Drain Current [A]
-50
-40
-30
-20
-10
0
10
Gate Voltage [V]

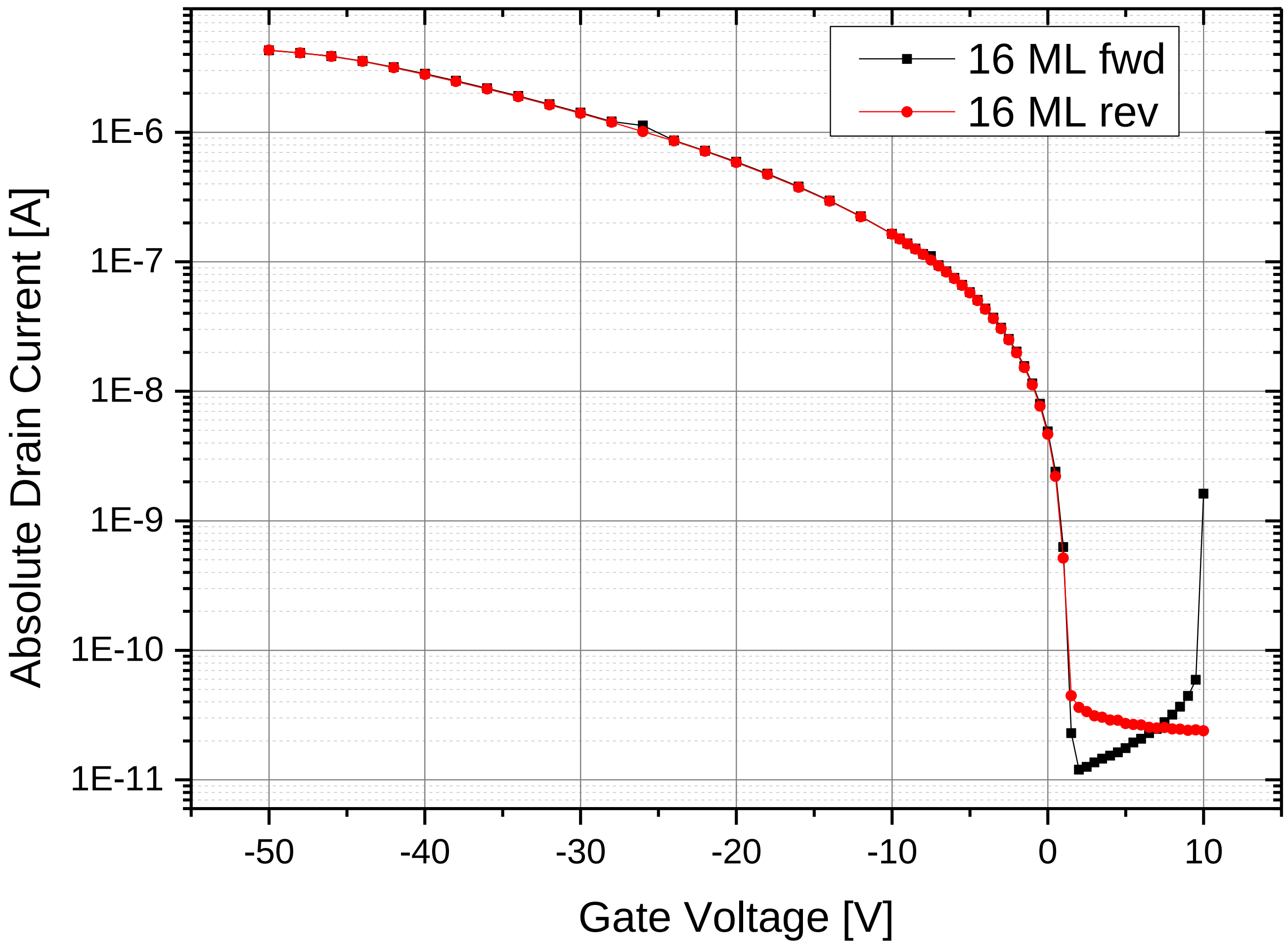

16 ML fwd
16 ML rev
1E-6
1E-7
1E-8
1E-9
1E-10
1E-11
Absolute Drain Current [A]
-50
-40
-30
-20
-10
0
10
Gate Voltage [V]

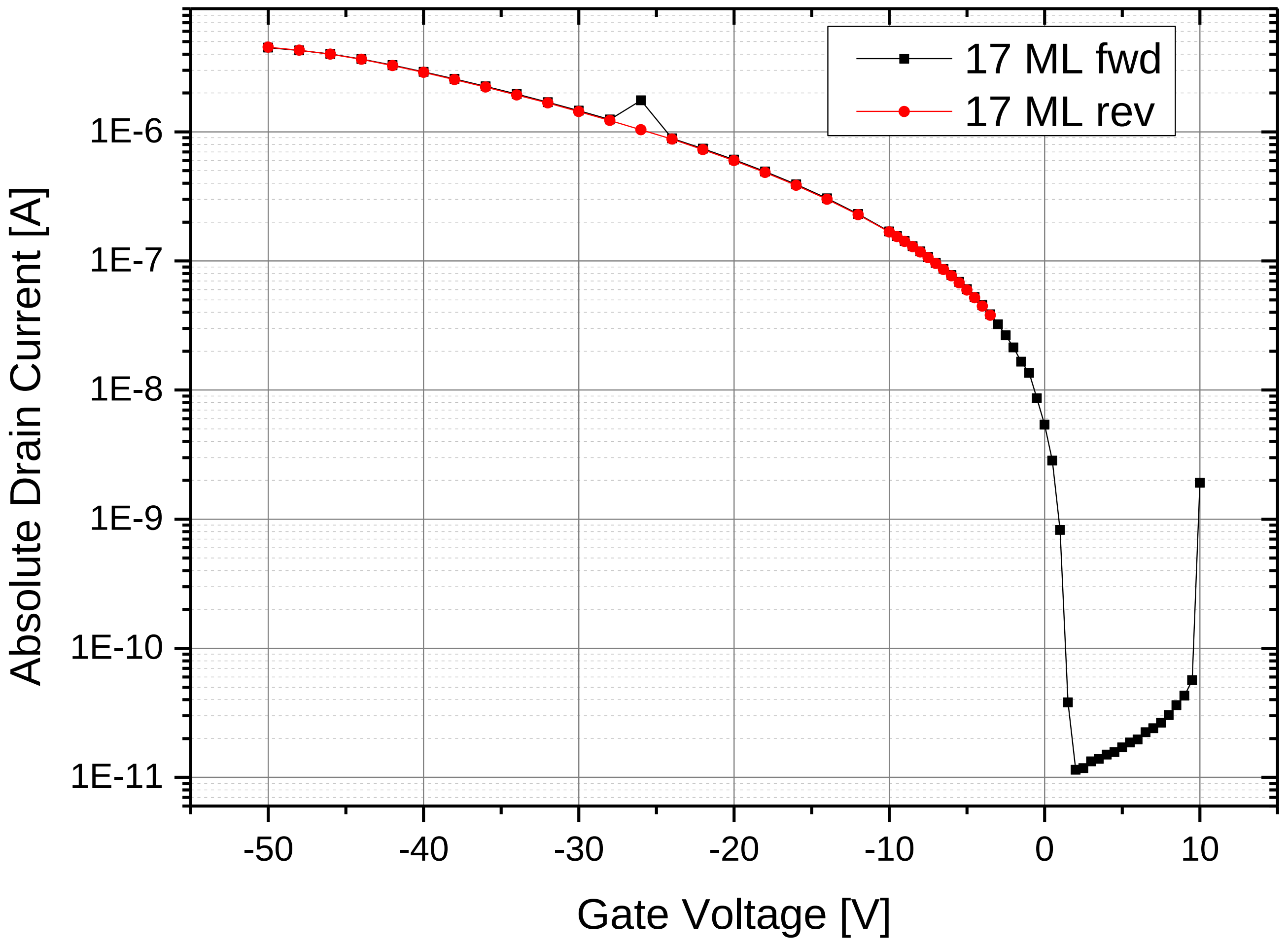

17 ML fwd
17 ML rev
1E-6
1E-7
1E-8
1E-9
1E-10
1E-11
Absolute Drain Current [A]
-50
-40
-30
-20
-10
0
10
Gate Voltage [V]

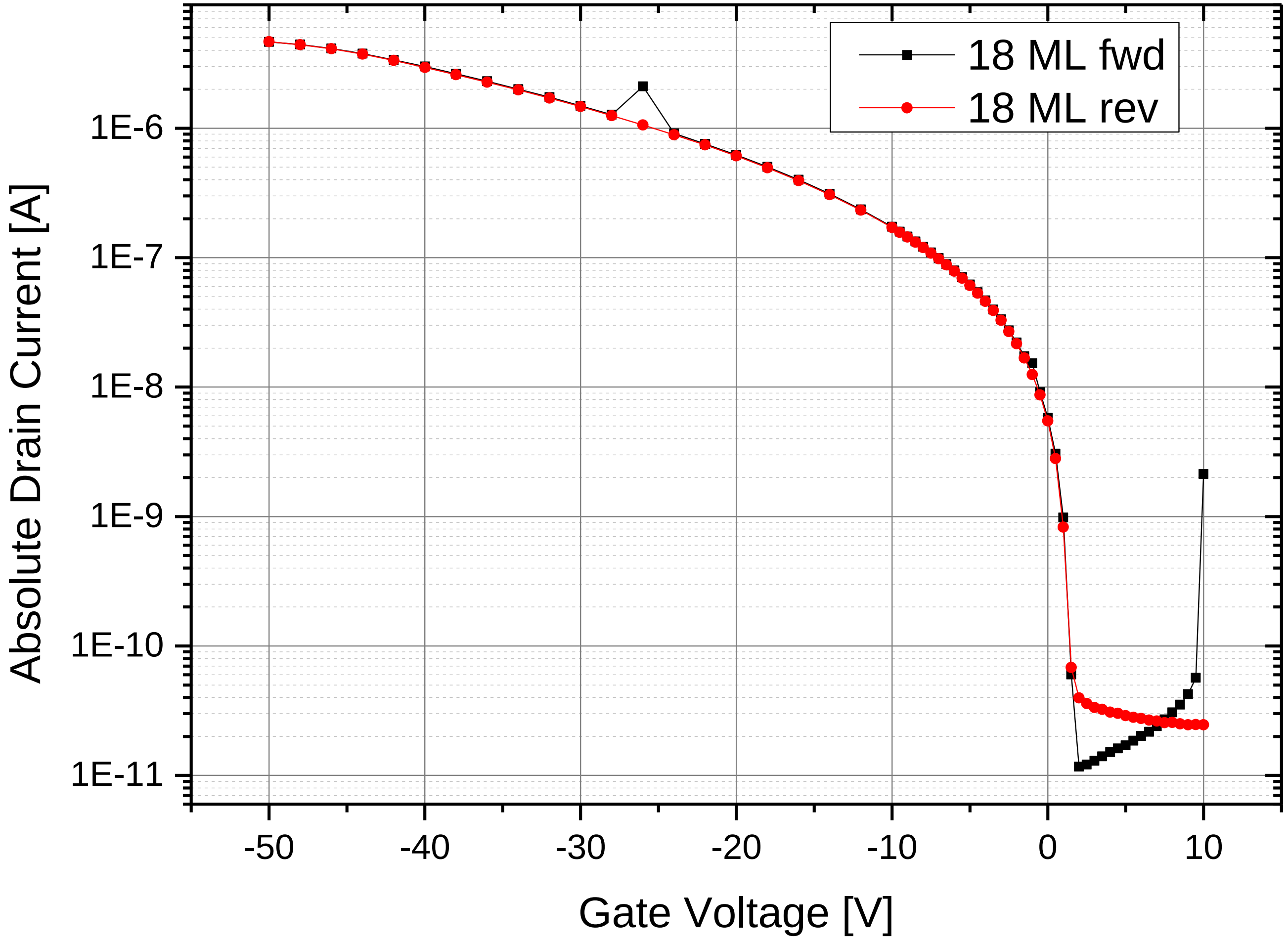

18 ML fwd
18 ML rev
Absolute Drain Current [A]
1E-6
1E-7
1E-8
1E-9
1E-10
1E-11
-50
-40
-30
-20
-10
0
10
Gate Voltage [V]

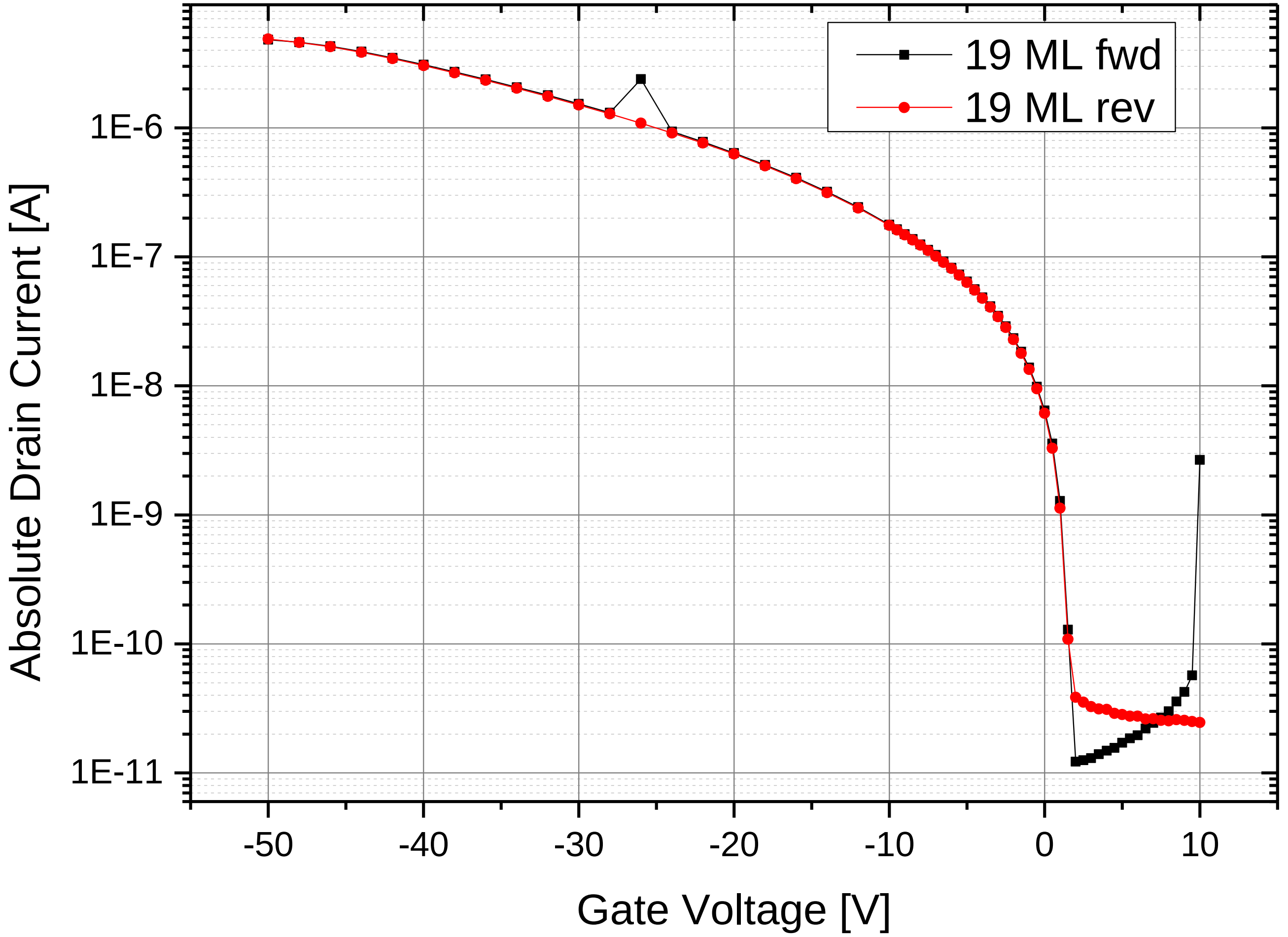

19 ML fwd
19 ML rev
1E-6
1E-7
1E-8
1E-9
1E-10
1E-11
Absolute Drain Current [A]
-50
-40
-30
-20
-10
0
10
Gate Voltage [V]

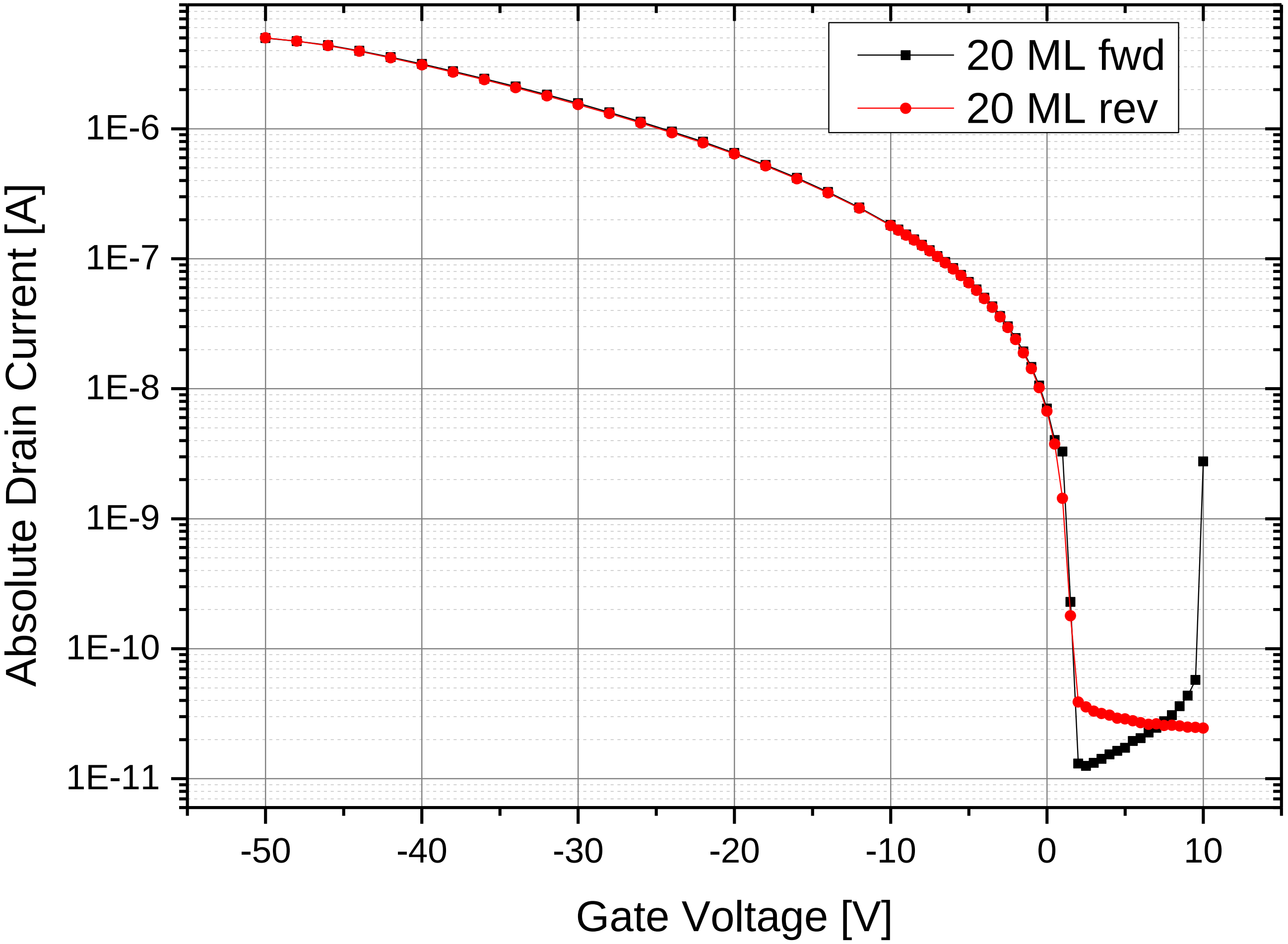
20 ML fwd
20 ML rev
1E-6
1E-7
1E-8
1E-9
1E-10
1E-11
Absolute Drain Current [A]
-50
-40
-30
-20
-10
0
10
Gate Voltage [V]

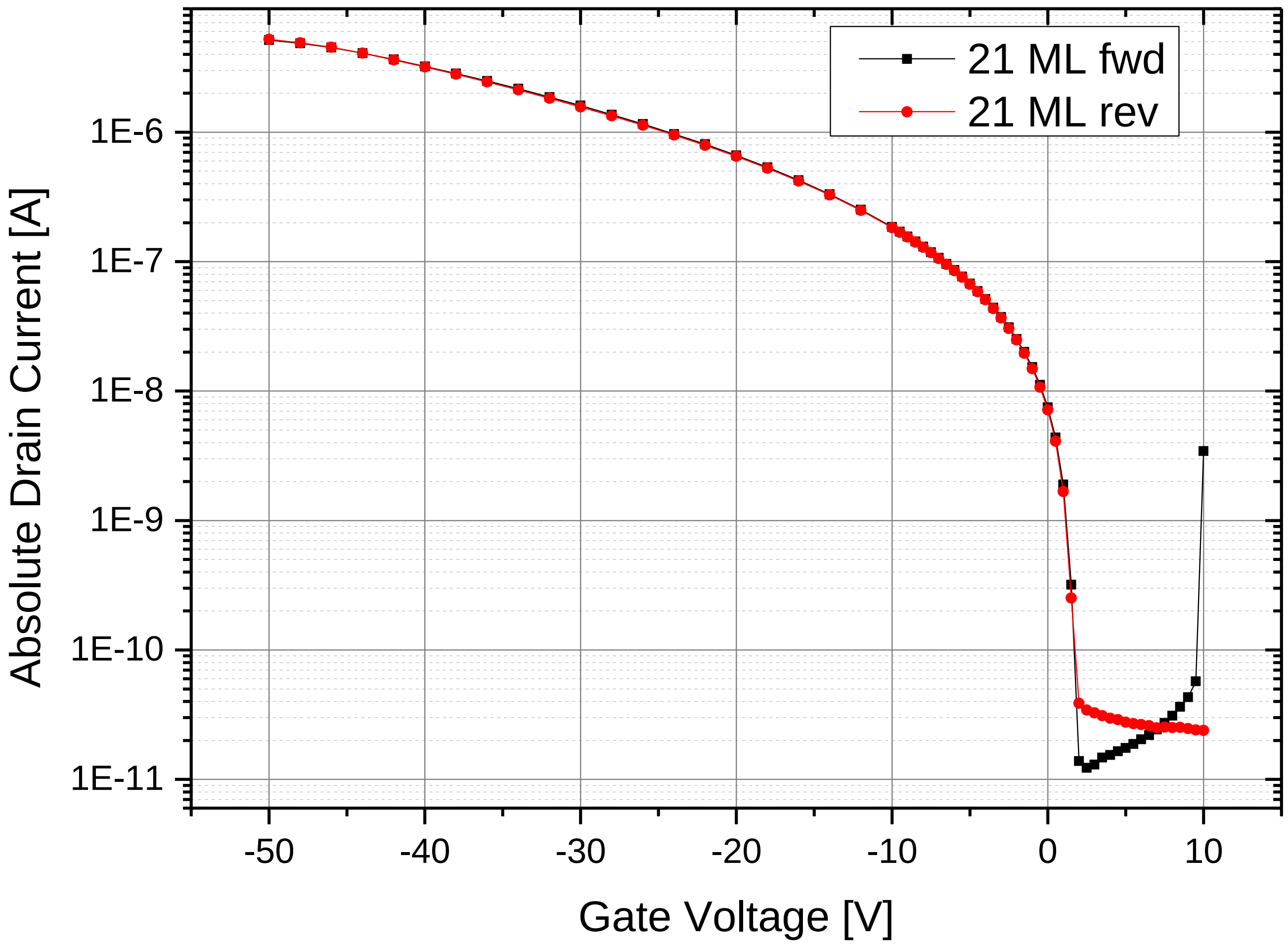

21 ML fwd
21 ML rev
1E-6
1E-7
1E-8
1E-9
1E-10
1E-11
Absolute Drain Current [A]
-50
-40
-30
-20
-10
0
10
Gate Voltage [V]

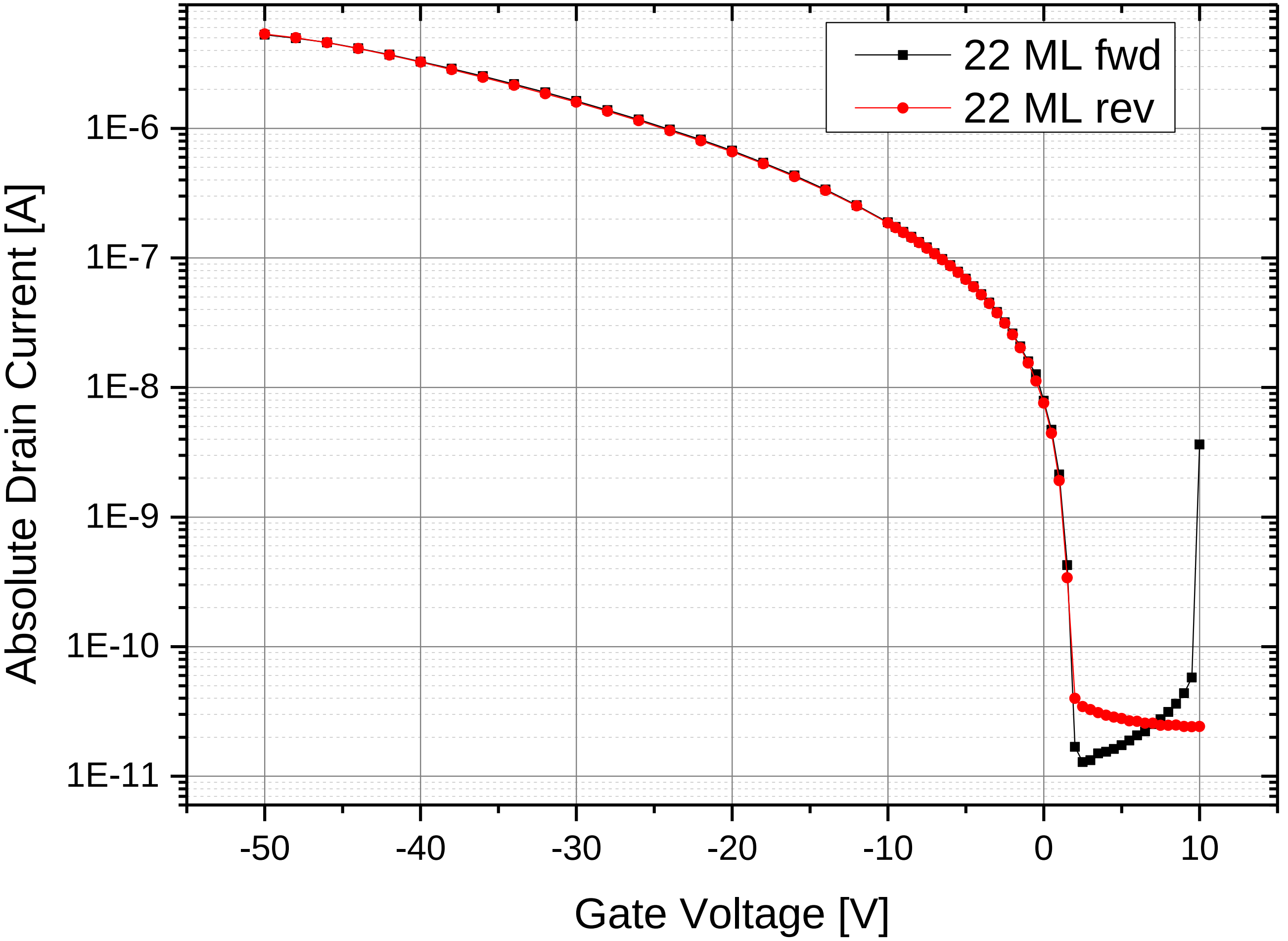

22 ML fwd
22 ML rev
Absolute Drain Current [A]
1E-6
1E-7
1E-8
1E-9
1E-10
1E-11
-50
-40
-30
-20
-10
0
10
Gate Voltage [V]
